# Thiourea‐ and Amino‐Substituted Benzoxadiazole Dyes with Large Stokes Shifts as Red‐Emitting Probe Monomers for Imprinted Polymer Layers Targeting Carboxylate‐Containing Antibiotics

**DOI:** 10.1002/chem.202104525

**Published:** 2022-03-15

**Authors:** Virginia Valderrey, Kornelia Gawlitza, Knut Rurack

**Affiliations:** ^1^ Chemical and Optical Sensing Division Bundesanstalt für Materialforschung und -prüfung (BAM) Richard-Willstätter-Straße 11 12489 Berlin Germany

**Keywords:** anion recognition, antibiotics, benzoxadiazole dyes, charge transfer, fluorescence, molecular imprinting

## Abstract

Bifunctional fluorescent molecular oxoanion probes based on the benzoxadiazole (BD) chromophore are described which integrate a thiourea binding motif and a polymerizable 2‐aminoethyl methacrylate unit in the 4,7‐positions of the BD core. Concerted charge transfer in this electron donor‐acceptor‐donor architecture endows the dyes with strongly Stokes shifted (up to >250 nm) absorption and fluorescence. Binding of electron‐rich carboxylate guests at the thiourea receptor leads to further analyte‐induced red‐shifts of the emission, shifting the fluorescence maximum of the complexes to ≥700 nm. Association constants for acetate are ranging from 1–5×10^5^ M^−1^ in acetonitrile. Integration of one of the fluorescent probes through its polymerizable moiety into molecularly imprinted polymers (MIPs) grafted from the surface of submicron silica cores yielded fluorescent MIP‐coated particle probes for the selective detection of antibiotics containing aliphatic carboxylate groups such as enoxacin (ENOX) at micromolar concentrations in highly polar solvents like acetonitrile.

## Graphical abstract

**Signalling in the red**, when antibiotics are bound to novel dual functional donor‐acceptor‐donor‐substituted benzoxadiazole probes with unprecedented large Stokes shifts, containing a benzoylthiourea receptor for carboxylate binding and a tertiary amine‐appended methacrylate linker for covalent matrix incorporation. The selective binding of a model antibiotic enoxacin is achieved by the integration of the polymerizable probes into molecularly imprinted polymer (MIP) shells on submicron silica particles, the dyes retaining their red‐shifted emission based on two concerted intramolecular charge transfer processes.

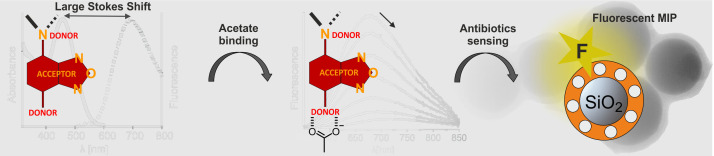

## Introduction

The recognition of oxoanions is of great interest in the field of supramolecular chemistry, due to their biological, industrial, and environmental relevance, in particular in the context of devising new anion‐responsive elements for a future integration into chemical sensors.^[1]^ Among receptors for polyatomic anions, those designed to target carboxylates^[2]^ are particularly relevant since such anions play a crucial role in metabolic processes.^[3]^ Carboxylates are also typical functional groups of many drugs such as antibiotics.^[4]^ For the optical detection of anions, fluorescent organic receptors are commonly used because they provide low detection limits and allow for a real‐time response. In the design of fluorescent probes for carboxylates of biological or environmental interest, there are usually two important considerations: anion binding should be compatible with (protic) polar media and fluorescence emission should ideally occur within the red to near‐infrared (NIR) spectral range, the so‐called “(first) biological window”.^[5]^ This is important since in this spectral window, autofluorescence from samples is usually minimal while water or tissue do not show sizable absorption.^[6]^ NH groups, especially when combined with other electron‐withdrawing functional groups such as carbonyl or thiocarbonyl, are ideally suited to target carboxylate moieties on potential analyte molecules, in particular, when arranged in a Y‐shaped motif such as a urea or thiourea because such a motif ideally matches the planar Y‐shaped structure of a carboxylate, qualifying for directed hydrogen bonding (H bonding) interactions.^[7]^ Directed H bonding is important when a recognition event should trigger an optical or electrochemical signal, because it induces defined electron density changes in the electronic π system of a host molecule. Thioureas, being more H‐acidic than ureas, have been coupled with different chromophores acting as recognition moieties for anions in polar media.[^2a^, ^2b^, ^2c^, ^8^] However, in most of the examples reported, the fluorescence emission maximum is not higher than 500 nm, still far away from the desired first biological window.

The fluorescent probes for anions described up to now mainly focus on controlling photoinduced electron transfer (PET)[^2a^, ^2c^, ^2d^, ^8a^] or exciplex^[9]^ processes. However, most of these probes limit their response to changes in fluorescence emission intensity, quenching or enhancement, but do not show spectral shifts of the emission bands upon anion binding. Alternative strategies involving the reconfiguration of multiple intra‐ and intermolecular H bonds or excited‐state proton transfer also usually operate in the same way.[^2e^, ^10^] This can severely limit their applicability especially in applications that utilize ratiometric measurements. An approach to obtain spectral shifts for fluorescent anion probes is their operation through an intramolecular charge transfer (ICT) mechanism.[^8b^, ^11^] For this purpose, an anion binding unit is commonly introduced as the donor (D) or the acceptor (A) site into a donor‐acceptor (D−A) system.[^2b^, ^12^] If the anion binding unit is acting as the donor site of a D−A system, a desired red‐shift in fluorescence emission results upon anion binding, because the binding of the anionic species increases the electron donating character of that part of the ICT system.^[12b]^ As a specific type of an ICT process, twisted intramolecular charge transfer (TICT) can occur in molecules that possess D and A units which are only separated by a single bond and for which a charge‐separated excited CT state can be stabilized by internal decoupling through adoption of a perpendicular orientation, commonly resulting in a strongly red‐shifted emission.^[13]^ Largely Stokes shifted emission bands are desirable as they render signal acquisition even with low‐cost instrumentation straightforward, reduce self‐quenching effects and increase the signal‐to‐noise ratio.^[14]^ However, examples of fluorescent anion receptors based on a twisted intramolecular charge transfer (TICT) process are scarce[^2g^, ^15^] and TICT states are known to be frequently non‐emissive.^[13b]^

Based on our experience in achieving highly emissive, red‐shifted CT signalling,^[16]^ we opted for a donor‐acceptor‐donor concept in the design of the title compounds of this work. Because the final aim is the matrix integration of such probes through co‐polymerization, we did not rely on our earlier concept based on styryl base‐type dyes but reduced the size of the fluorophore, which is important for operation in confined matrices,^[17]^ and avoided chromophoric C=C double bonds, being problematic for co‐polymerization. Having in mind the beneficial characteristics of conventional push‐pull benzoxadiazole (BD) dyes established by others^[18]^ and us,^[19]^ we elaborated the concept in this work by developing a family of fluorescent anion probes that combine concerted CT processes in a thiourea‐ (as D_1_) and amino‐ (as D_2_) functionalized BD (A) D_1_−A−D_2_ chromophore, fusing both units D_1_ and D_2_ directly to the same aromatic A unit. The concerted CT should thus proceed along both routes D_1_→A←D_2_. In case of anion binding, analyte‐induced modulation of D_1_ would thus reinforce the combined CT processes, leading to bathochromic shifts.

The simple, rapid and selective (or class‐selective) detection of antibiotics is important since their residues are often unstable and decay with time to other more stable chemical species with potential environmental and health impact,^[20]^ necessitating in particular the development of methods that allow for antibiotics detection close to their point‐of‐use. Moreover, the presence of antibiotics in the environment is known to be associated with the development of antibiotic resistance.[^20^, ^21^] Therefore, several approaches to antibiotics detection that can be developed into onsite analytical methods have been realized in the past, involving molecularly imprinted polymers (MIPs),^[22]^ antibody‐ and other biomacromolecule binder‐functionalized nanoparticles,^[23]^ metal‐organic frameworks,^[24]^ polymers as sensor arrays^[25]^ and multiplexed aptasensors.^[26]^ Fluorescent MIPs for the detection of antibiotics have been described using graphene quantum dots (GQD)^[27]^ or fluorescein^[28]^ as the fluorescent units to interact with the target analytes. In these cases, the fluorescence emission changes are related to the electrostatic interaction between the GQD or fluorescein and the templates but provide only small intensity changes in the fluorescence emission spectrum. Here, in addition to the newly developed bifunctional probe architecture, we present the incorporation of one of the title dyes into fluorescent MIP layers coated onto silica cores for the selective recognition of enoxacin in polar solvents in the micromolar concentration, presenting a promising approach toward future MIP‐based sensors.

## Results and Discussion

As has been recently demonstrated, the benzoxadiazole (BD) framework does not only possess favorable spectroscopic properties, but also is the synthesis of functional BD dye derivatives considerably straightforward.[^18^, ^19^] Accordingly, access to the title dye family was also possible through a three‐step procedure starting from commercial precursors.

### Synthesis

**Scheme 1:**
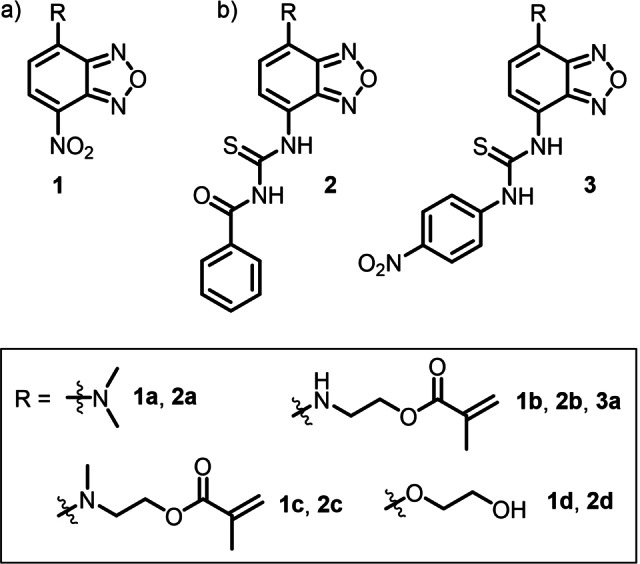
Molecular structures of benzoxadiazoles containing a) a nitro group as second acceptor group fused to the BD acceptor (**1**) and b) benzoyl and nitrophenyl thioureas (**2** and **3**) as donating D_1_ groups as well as R, representing the electron‐donating amino or alkoxy group D_2_.

The synthesis of the fluorescent anion probes first involved the electrophilic aromatic substitution of commercially available 4‐chloro‐7‐nitrobenzoxadiazol with dimethylamine or 2‐aminoethyl methacrylate hydrochloride in the presence of a base, to obtain **1** **a** and **1** **b**, respectively (Schemes 1 and S1, Supporting Information). Subsequently, methylation of **1** **b** afforded the dialkyl derivative **1** **c**. The alkoxy nitro‐BD derivative **1** **d** was obtained following described procedures.^[29]^

Nitrobenzoxadiazoles **1** were further reduced to the amino analogues with iron powder and acetic acid as solvent. The reaction of the amino BDs with either benzoyl isothiocyanate or 4‐nitrophenyl isothiocyanate provided the thiourea benzoxadiazoles **2** and **3**, respectively, in good yields (see Experimental Section and Supporting Information for more details).

### Spectroscopic studies

**Figure 1:**
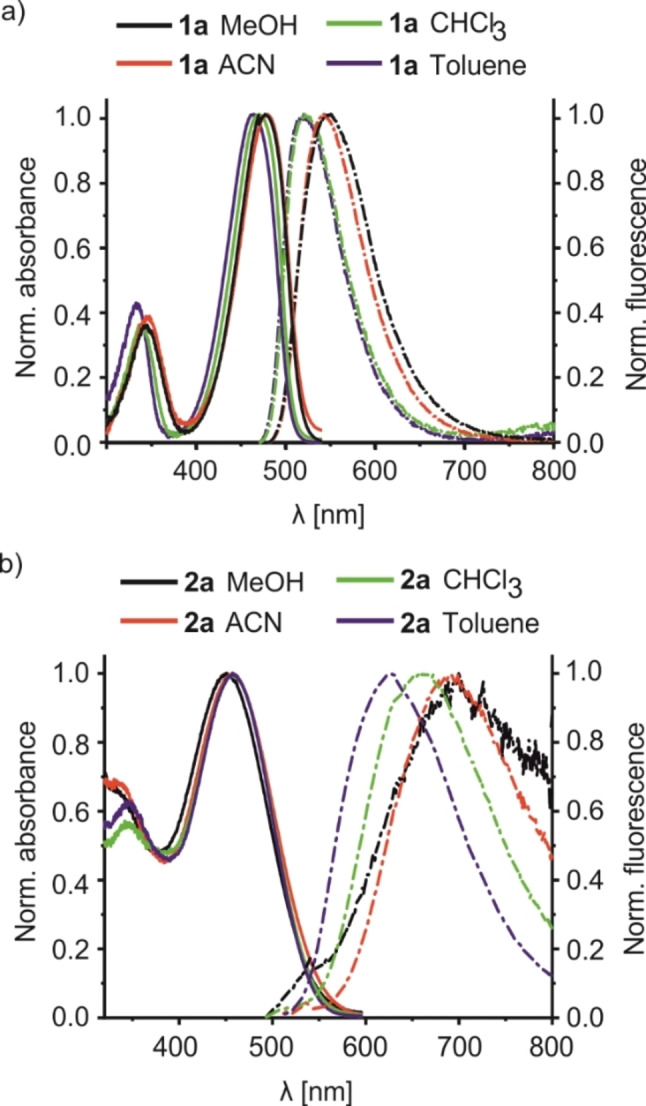
Normalized absorption and emission (*λ*_ex_=465 nm) spectra of a) **1** **a** (*c*_**1a**_=4.0×10^−6^ M) and b) **2** **a** (*c*_**2a**_=1.0×10^−5^ M) in solvents of different polarity.

Our initial studies focused on understanding the nature of the fluorescence emission of the benzoxadiazole family comprising dyes **1**, **2** and **3**. As has been reported before,^[30]^ dye **1** **a** shows an absorption spectrum centered at ca. 465 nm, slightly shifting to longer wavelengths as the solvent polarity increases. Consistent with an ICT process in such so‐called “push‐pull” dyes, the excited CT state usually has a higher dipole moment than the ground state, leading to a more pronounced positive solvatochromism in fluorescence, here with Stokes shifts of 56 nm in toluene and 63 nm in acetonitrile (Figure 1a). In the case of **1** **a**, the aromatic BD acceptor carries an amino donor and a nitro group as a second acceptor in direct π conjugation, exemplifying a conventional D−A dye. The analogous dye **1** **b**, carrying a secondary amino group instead of a tertiary, also shows a positive solvatochromism with somewhat blue‐shifted absorption bands yet slightly larger Stokes shifts of ca. 75 nm, tentatively ascribed to the slightly weaker electron donor strength yet higher coplanarity of the secondary amine unit NHR (see Figure S7 vs. S6). The replacement of the amino for the still weaker electron‐donating ethoxy group in **1** **d** further reduces the D−A character of the dyes, providing absorption spectra which are ca. 60 nm blue‐shifted as compared with the amino substituted counterparts. However, for **1** **d**, weak fluorescence emission was only recorded in toluene, with an unusually large Stokes shift of 167 nm, the origin of which is currently still unclear (Figure S8).

In contrast to **1** **a** and **1** **b**, which follow a D−A architecture, dyes **2** are designed as D_1_−A−D_2_ chromophores with the BD core acting as the acceptor and thiourea and amino groups as the donors. In the case of **2** **a**, a qualitatively similar behaviour as for **1** **a** is observed only that the Stokes shifts are much larger, ranging from 155 nm in toluene to 255 nm in MeOH (Figure 1b). This distinctly higher solvatochromism is ascribed to the concerted ICT in the D−A−D dye. To the best of our knowledge these Stokes shifts are among the largest described for red/NIR emitting dyes.[^14b^, ^18b^, ^31^] Again, going from a tertiary amine in **2** **a** to a secondary amine in **2** **b** leads to hypsochromic displacements of the absorption (19 nm) and emission (15 nm) bands in toluene (Figure S9), reflecting the situation of **1** **a** vs. **1** **b**. Introducing an asymmetric tertiary amine as in **2** **c** leads again to similar spectroscopic characteristics as observed for **2** **a** (Figure S9). Furthermore, also for this series of D−A−D dyes, substitution of the electron‐donating amino group for an alkoxy substituent (**2** **d**) yields a dye with strongly blue‐shifted absorption and emission bands, emitting outside of the desired biological window.

To verify that the exceptionally red‐shifted bands of **2** **a**, **2** **b** and **2** **c** are due to an ICT process and not for instance due to excimer formation, concentration‐dependent studies were performed with **2** **b**, yielding identical spectral shapes in the concentration range <2.5×10^−5^ M, thus excluding the latter.

The fluorescence quantum yields (*Φ*_f_) for **2** **b** and **2** **c** were determined to 0.004 and 0.05 in ACN, respectively. Altogether, the results of the spectroscopic studies support our design considerations that thiourea‐substituted benzoxadiazoles functionalised with an amino group as D_2_ are promising fluorescent dyes for red‐emissive anion probes with large Stokes shifts. Among them, especially **2** **c**, with the highest fluorescence quantum yield, seems to be the most potent candidate for sensor material development.

### Binding studies in solution

After evaluating the spectroscopic characteristics of thiourea benzoxadiazoles **2**, we proceeded to study their binding behaviour towards carboxylates in different solvents by UV/Vis absorption and fluorescence spectroscopy.

**Figure 2:**
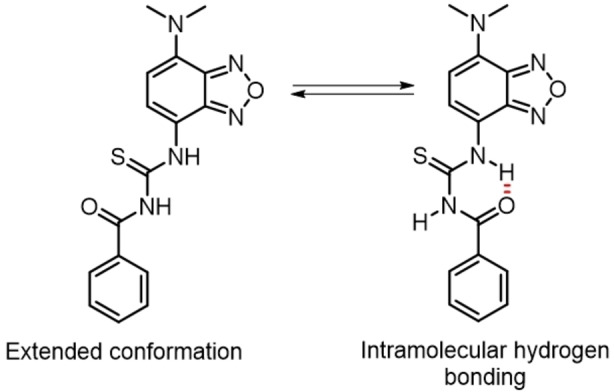
Conformational equilibrium between the extended and the intramolecularly hydrogen‐bonded conformations of **2** **a**.

The addition of more than 1000 equivalents of tetrabutylammonium acetate (AcO‐TBA) to a 1.2×10^−5^ M chloroform solution of **2** **a** did not produce any spectroscopic change in absorption (Figure S10a). We tentatively ascribe this unresponsiveness to the carbonyl subunit of the benzoyl group attached to the thiourea being involved in an intramolecular hydrogen bonded conformation of **2** **a** that prevents formation of the Y‐shaped H bonding motif and thus binding of acetate (Figure 2a).^[32]^ To confirm this hypothesis, we synthesized the nitrophenyl analogue **3** **a** where the formation of an intramolecular hydrogen bond with the thiourea NH is not possible. Indeed, upon addition of only stoichiometric amounts of AcO‐TBA to a 2.5×10^−5^ M chloroform solution of **3** **a**, binding‐induced changes in the absorption spectrum were observed, which indicates the expected tight binding of a thiourea unit substituted with two aromatic electron acceptor units (Figure S10b). When the benzoyl derivative **2** **a** is dissolved in polar solvents, like ACN, such intramolecular hydrogen bonding is less expected, the solvation by the highly dipolar solvent stabilizing better the extended conformation of the dye.

**Figure 3:**
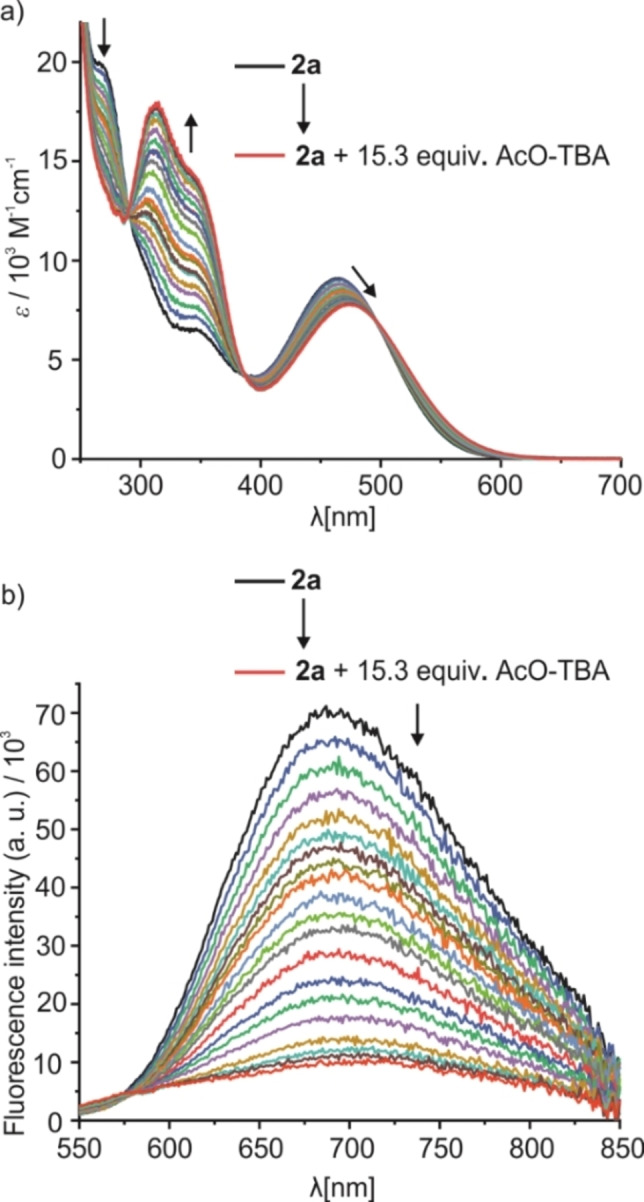
a) Absorption and b) emission (*λ*_ex_=499 nm) spectra of **2** **a** (*c*_**2a**_=1.1×10^−5^ M) upon addition of up to 15.3 equiv. of AcO‐TBA in ACN.

With these results in mind, we evaluated the binding behavior of **2** **a** and AcO‐TBA in ACN (Figure 3). Upon the addition of AcO‐TBA to a solution of **2** **a** the band at 275 nm decreased, suggesting that the NHs adjacent to the benzoyl groups are being involved in the binding event. Moreover, the band at 450 nm corresponding to the main D−A−D chromophore shows a bathochromic shift with an isosbestic point at 499 nm. As explained above, when sketching our design considerations, this effect can be rationalized by the reinforced CT process because of the binding of an electron‐rich anionic species at the thiourea receptor as D_1_. Similar spectral features were observed for the interaction of **2** **a** and AcO‐TBA in other medium polar aprotic yet weakly hydrogen bond accepting solvents such as THF and ethyl acetate (see Figure S10c and d). The binding constant for the interaction was calculated to be *K*_**2a**@AcO‐TBA_=1.0×10^5^ ±6.8×10^3^ M^−1^ for a 1 : 1 binding model in ACN, which agrees well with values reported for anion probes containing a thiourea functionality in this solvent.^[33]^

**Figure 4:**
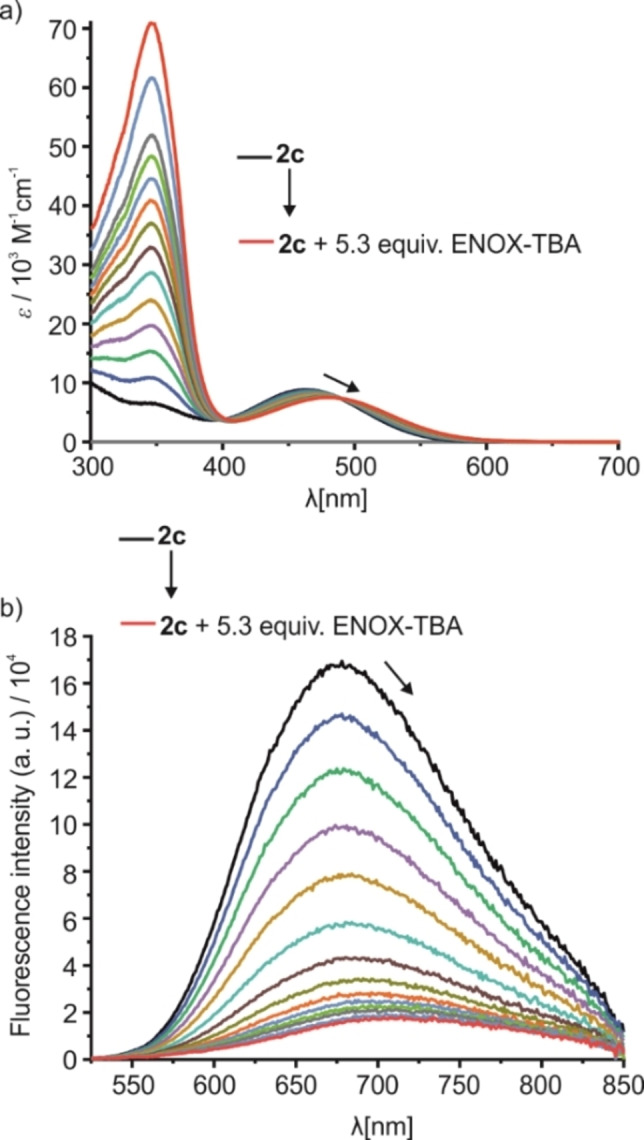
a) Absorption and b) emission (*λ*_ex_=480 nm) spectra of **2** **c** (*c*_**2c**_=1.1×10^−5^ M) upon addition of up to 5.3 equiv. of ENOX‐TBA in ACN.

Fluorescence emission spectra were also recorded during the titration and an effective quenching of the fluorescence was observed by exciting at the isosbestic point corresponding to the titration in absorbance. To study the possible influence of the substituents in *para*‐position to the thiourea, in terms of binding strength and effectiveness of the fluorescence changes, we performed similar titration experiments with **2** **b** and **2** **c**. The titration with structurally analogous **2** **c**, which contains an ethyl‐appended polymerizable unit instead of one methyl group, gave a rather similar binding constant for acetate, *K*_**2c**@AcO‐TBA_=1.1×10^5^±3.1×10^4^ M^−1^ (determined by UV/Vis titration experiments). In this case, an isosbestic point was observed at 488 nm (Figure S12a). Exciting at this wavelength, a fluorescence emission at 675 nm was recorded that was red‐shifted by up to 45 nm and quenched upon addition of AcO‐TBA (Figure S12b). Moreover, subjecting **2** **b** with a secondary amine in the 7‐position of the BD core to a respective titration experiment, again rather similar results were obtained (*K*_**2b**@Ac‐TBA_=9.8×10^4^ ±2.6×10^4^ M^−1^; Figure S13). The alkoxy derivative **2** **d** showed less pronounced spectral changes in absorption upon the addition of AcO‐TBA, the spectra being generally significantly blue‐shifted because of the lower donor strength of the alkoxy group as D_2_ (Figure S14a). The magnitude of the fluorescence emission response as such was also smaller for **2** **d** compared with **2** **a**–**c**, disqualifying this approach for further sensor material integration (Figure S14b). Based on these results, we concluded that **2** **c** indeed qualifies as the best candidate in terms of binding affinity and fluorescence response to proceed toward an efficient sensor material for aliphatic carboxylate‐containing compounds. In this regard, we chose the antibiotic enoxacin (ENOX) as relevant target compound and studied the response of **2** **c** toward the tetrabutylammonium salt of ENOX (ENOX‐TBA). Again, UV/Vis and fluorescence titrations in ACN were performed (Figure 4a and b).

As expected, the addition of ENOX‐TBA to a solution of **2** **c** in ACN produced a bathochromic shift of the lowest energy absorption band with an isosbestic point at 480 nm. The binding constant for this interaction was calculated to be *K*_**2c**@ENOX‐TBA_=4.2×10^5^±1.8×10^5^ M^−1^ in ACN, which is higher than for acetate, presumably due to the higher electron density of the carboxylate group in ENOX. A quenching of the fluorescence together with a 52 nm red shift of the emission band was observed for this titration.

### Molecularly imprinted polymers

**Figure 5:**
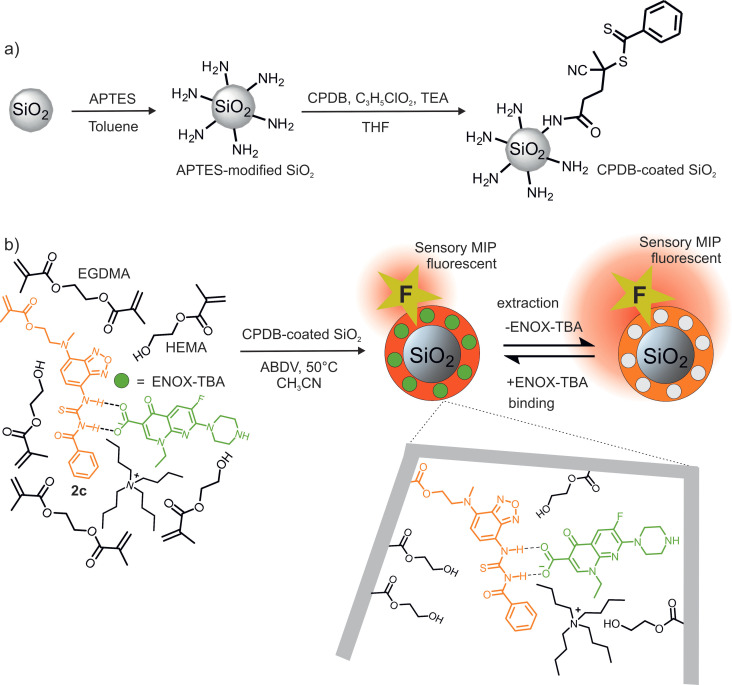
Schematic of the route to ENOX‐TBA‐imprinted polymer shells on SiO_2_ core particles consisting of: a) preparation of CPDB‐coated SiO_2_ microparticles followed by b) RAFT polymerization utilizing H bonding between **2** **c** and ENOX‐TBA, allowing for its incorporation into the polymer network. Subsequent extraction of ENOX‐TBA yields the fluorescent sensory MIP. APTES: (3‐aminopropyl)triethoxysilane, CPDB: 4‐cyano‐4‐(thiobenzoylthio)pentanoic acid, TEA: triethylamine.

After proving the detection ability of probe **2** **c** in ACN solution towards enoxacin, we proceed to incorporate the probe monomer into polymeric matrices for sensor material development. Our aim was to produce thin molecularly imprinted polymer (MIP) shells on submicron silica (SiO_2_) particles since these can be used in bead‐based assays or integrated into sensory membranes.^[19a]^ RAFT (reversible addition‐fragmentation chain‐transfer) polymerization was chosen as the polymerization technique which would provide homogeneous thin polymer networks with high binding capabilities and fast response times.[^19a^, ^34^] Monodisperse SiO_2_ microparticles of 316±26 nm diameter were used as inert support of the polymer network. The SiO_2_ particles were initially functionalized with 3‐aminopropyltriethoxysilane (APTES) followed by coupling with 4‐cyano‐4‐(thiobenzoylthio)pentanoic acid (CPDB) as RAFT agent according to described procedures (see Figure 5 and Supporting Information for details).^[19a]^ Based on the amount of sulphur determined by elemental analysis (0.0014 g g^−1^ of particles) and the specific surface area of the silica particles determined from porosimetry (15.59 m^2^ g^−1^ of particles), the density of RAFT groups on the particle surface was calculated to be 0.84 molecules nm^−2^. As has proven to be most efficient in earlier works on fluorescent MIPs,[^19^, ^35^] the composition and ratio of co‐monomers and cross‐linkers were screened in spectroscopic prepolymerization studies. As a result, a mixture of **2** **c** and ENOX‐TBA as template in equimolar amounts together with the co‐monomer 2‐hydroxyethyl methacrylate (HEMA) and the cross‐linker ethylene glycol dimethacrylate (EGDMA) in a 60 : 40 ratio, respectively, showed adequate template‐dye complex formation and hence was used for the MIP synthesis (Figure 5; see Experimental Section and Supporting Information for additional details). The use of HEMA, a polar structural monomer, and EGDMA,^[36]^ a commonly used cross linker, to form polar polymer matrices allowed us to obtain MIPs with a good dispersibility in ACN. The different synthesis and functionalization steps of the MIP particles led to changes in the particle surface properties, as revealed by zeta potential measurements (Figure S15a). As expected in purified water at pH 6, silica particles yielded a negative surface charge due to the presence of silanol groups. APTES modification introduced amino groups on the surface leading to a net positive surface charge, whose magnitude was reduced after reaction with CPDB to graft RAFT groups onto the surface. Synthesis of a crosslinked polymer MIP layer on the particle surface covered the remaining unconverted amino groups on the surface, with a nearly neutral charge being obtained for the MIP particles. TGA measurements indicated mass loss from combustion of organic groups that were adsorbed or covalently attached on the silica surface indicating a thick polymer shell in case of MIPs (Figure S15b).

**Figure 6:**
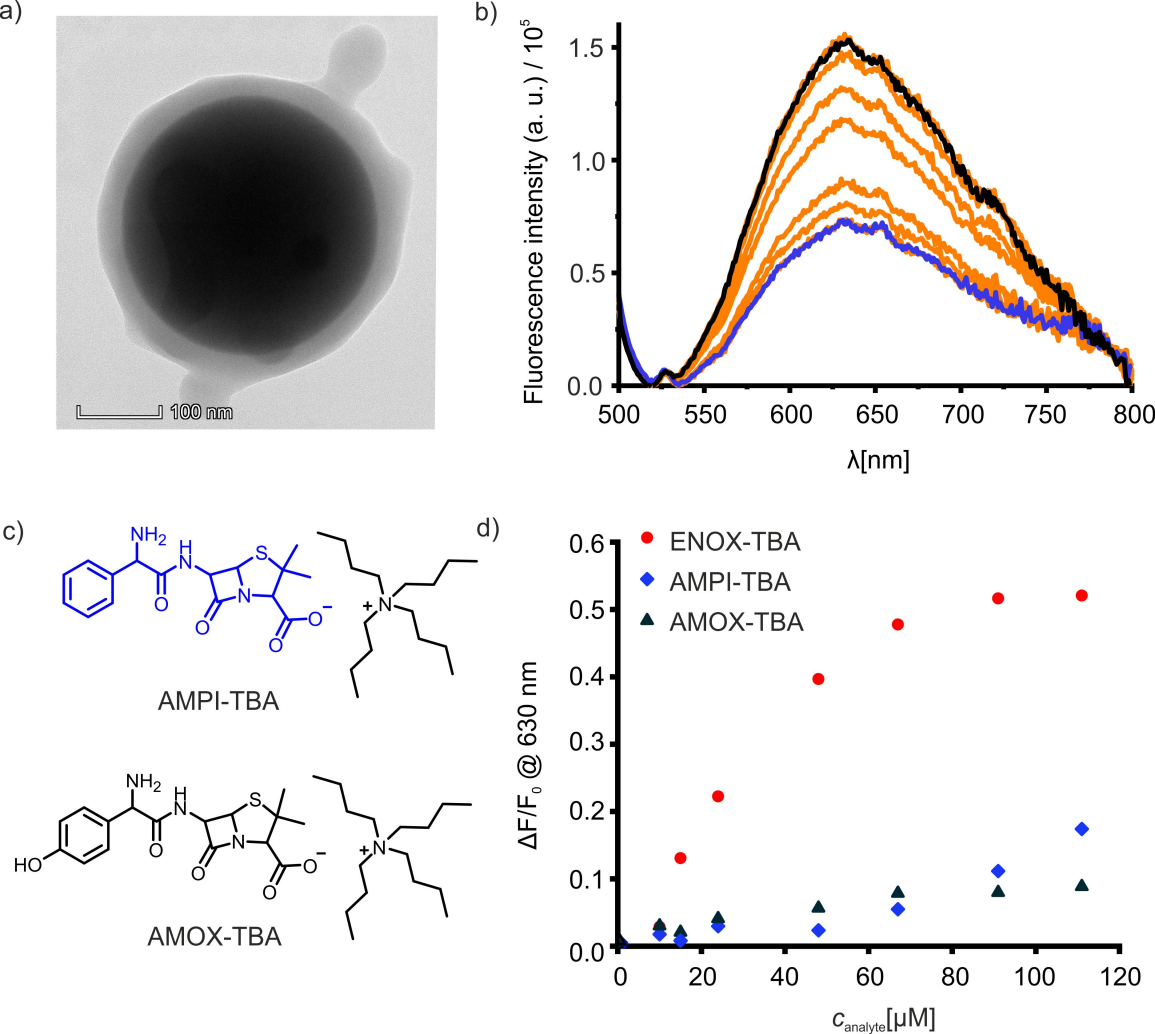
a) TEM image of an ENOX‐TBA imprinted core‐shell silica particle. Scale bar is 100 nm. b) Fluorescence emission (*λ*_ex_=480 nm) spectra of the sensory MIP (see Figure 5) in the absence (black line) and in the presence of up to 111 μM ENOX‐TBA (blue line). c) Chemical structures of the antibiotics ampicillin and amoxicillin as their tetrabutylammonium salts. d) Normalized fluorescence changes (Δ*F*/*F*_0_ with Δ*F*=*F*_0_−*F*) against concentration of analyte: ENOX as red circles, ampicillin as blue diamonds and amoxicillin as black triangles, all of them detected as the TBA salts (total relative errors are 3.3 % as described in Supporting Information).

TEM images of the MIP shells on the silica cores showed a homogeneous polymer layer with a thickness of 32±6 nm (Figures 6a and S16). Absorption and fluorescence spectroscopy were used to prove the incorporation of **2** **c** into the polymer network on the silica cores, UV/Vis measurements revealing a content of co‐polymerised fluorescent probe monomer into the MIP layer of ca. 9.6×10^−26^ mol nm^−3^ (Figure S17). This successful incorporation shows that **2** **c** remains stable under polymerization conditions. The fluorescence band of a suspension of the core‐shell particles in ACN was centred at 630 nm. This hypsochromic displacement with respect to the maximum band position of neat **2** **c** in ACN can be ascribed to two factors, the confinement of the probe monomer in the network, which does not allow for full relaxation of the excited ICT state, and the changed microenvironmental polarity because of the interaction with a mixture of co‐monomers, cross‐linkers and solvent instead of the neat solvent as in the molecular interaction studies.

The sensing ability of the core‐shell particles was proven by the incremental addition of ENOX‐TBA to a suspension of the particles in ACN. A gradual quenching of the fluorescence emission band at 630 nm was observed until the virtual absence of a fluorescence signal at a concentration of 111 μM of ENOX‐TBA (Figure 6b). Kinetic studies revealed that complexation after diffusion of the analyte into the polymer matrix is completed 120 s after analyte addition. According to previous reports,[^19a^, ^37^] MIP matrices with thicknesses of only few nanometres enable such fast responses toward small‐molecule analytes.

The fluorescence response toward other relevant antibiotics containing carboxylic acid moieties was also tested, to evaluate the selectivity of the MIPs developed for ENOX. Fluorescence emission titrations with ampicillin (AMPI) and amoxicillin (AMOX) as the TBA salts were carried out in the same manner as before (Figures 6c, S18 and S19). After representing the normalized fluorescence changes vs. the concentration of the three analytes, we can conclude that, at low concentrations (<100 μM), there is a good discrimination between ENOX and the two competitors (Figure 6d), represented by high discrimination factors, [Δ*F*/*F*_0_]_ENOX_/[Δ*F*/*F*_0_]_analogue_, of 5 and 6 against AMPI‐TBA and AMOX‐TBA, respectively. Thus, the sensory MIP presented here qualifies as selective fluorescent sensor material for ENOX in polar solvents, showing fluorescence in a favourable spectral range and large Stokes shifts, ideal for simple optical detection setups.

## Conclusion

In conclusion, we have described the synthesis and spectroscopic evaluation of a new family of dyes derived from benzoxadiazoles. The benzoxadiazole core, acting as an acceptor unit, has been functionalized with two electron‐donating groups: a thiourea receptor and an amine, which can easily be equipped with a polymerizable unit for further covalent incorporation into a polymer matrix. The donor‐acceptor‐donor architecture guarantees that a concerted intramolecular charge transfer process is effective, leading to extraordinary large Stokes shifts so that these dyes emit in an application‐relevant wavelength range, that is, the so‐called biological window, already in medium to highly polar solvents.

The interaction between polymerizable fluorescent probes **2** with acetates as TBA salts in ACN revealed bathochromic shifts in both, absorption and especially fluorescence, together with a decrease in emission. Upon incorporation of the best performing probe monomer **2** **c** as a hydrogen bonded complex with enoxacin as the TBA salt into a molecularly imprinted polymer shell on submicron silica particles, these sensory particles could be used to rebind the analyte ENOX selectively in the micromolar concentration range. The spectroscopic characteristics of the probe monomer within the polymer matrix were studied and compared with the ones in solution. Although confinement leads to a certain hypsochromic shift, which is commonly expected for ICT active dyes, still the wavelength range of fluorescence is very advantageous for optical sensing devices. Current work in our laboratory is thus devoted to further device integration and improvement of the molecular probe platform.

## Experimental Section

### General information

Details on chemicals, instruments, methods and syntheses of precursors and dyes **1** **a**–**d**, **2** **d** and **3** **a** are given in the Supporting Information.

### Benzoxadiazole syntheses

2‐((7‐(3‐benzoylthioureido)benzo[c][1,2,5]oxadiazol‐4‐yl)(dimethyl) (**2** **a**). *N*,*N*‐dimethyl‐7‐nitrobenzo[c][1,2,5]oxadiazol‐4‐amine (**1** **a**) (0.145 g, 0.70 mmol) was dissolved in 4.0 ml of acetone. Benzoyl isothiocyanate (98.0 %) (0.095 ml, 0.70 mmol) was added and an orange precipitate was formed after 5 min. The precipitate was filtered to afford a red solid as the desired compound (0.11 g, 50 % yield). ^1^H NMR (400 MHz, CDCl_3_): *δ* (ppm)=13.18 (bs, 1H), 9.11 (bs, 1H), 8.49 (d, 1H), 7.94 (d, 2H), 7.66 (t, 1H), 7.56 (t, 2H), 6.09 (d, 1H), 3.34 (s, 6H). ^13^C NMR (100 MHz, CDCl_3_): *δ* (ppm)=176.74, 166.88, 146.97, 145.42, 138.09, 133.75, 131.55, 129.22, 127.58, 125.44, 113.54, 104.17, 42.05. HRMS (ESI+): m/z calculated for C_16_H_15_N_5_O_2_S ([M+H])^+^ 342.1025, found ([M+H])^+^ 342.1072. UPLC: *t*_R_=5.03 min (100 % peak area).

2‐((7‐(3‐benzoylthioureido)benzo[c][1,2,5]oxadiazol‐4‐yl)amino)ethyl methacrylate (**2** **b**). 2‐((7‐aminobenzo[c][1,2,5]oxadiazol‐4‐yl)amino)ethyl methacrylate (**II**) (0.102 g, 0.39 mmol) was dissolved in 5.0 ml of acetone. Benzoyl isothiocyanate (98.0 %) (0.056 ml, 0.39 mmol) was added and an orange precipitate was formed after 5 min. The precipitate was filtered to afford an orange solid as the desired compound (0.1 g, 60 % yield). ^1^H NMR (400 MHz, DMSO‐d6): δ (ppm)=12.84 (bs, 1H), 11.71 (bs, 1H), 8.02 (d, 1H), 7.99 (m, 2H), 7.66 (t, 1H), 7.55 (t, 2H), 7.36 (t, 1H), 6.32 (d, 1H), 6.02 (m, 1H), 5.65 (m, 1H), 5.27 (m, 1H), 4.33 (m, 2H), 3.62 (m, 2H), 1.86 (s, 3H). ^13^C NMR (100 MHz, DMSO‐d6): *δ* (ppm)=179.80, 169.01, 167.06, 147.02, 145.24, 136.19, 135.53, 133.63, 132.47, 129.58, 129.17, 128.89, 126.45, 112.84, 101.04, 62.97, 42.12, 18.44. HRMS (ESI+): *m*/*z* calculated for C_20_H_19_N_5_O_4_S ([M+H])^+^ 426.1236, found ([M+H])^+^ 426.1236. UPLC: *t*_R_=4.16 min (100 % peak area).

2‐((7‐(3‐benzoylthioureido)benzo[c][1,2,5]oxadiazol‐4‐yl)(methyl)amino)ethyl methacrylate (**2** **c**). 2‐((7‐aminobenzo[c][1,2,5]oxadiazol‐4‐yl)(methyl)amino)ethyl methacrylate (**III**) (0.098 g, 0.35 mmol) was dissolved in 3.0 ml of acetone. Benzoyl isothiocyanate (98.0 %) (0.048 ml, 0.35 mmol) was added and an orange precipitate was formed after 5 min. The precipitate was filtered to afford an orange solid as the desired compound (0.090 g, 60 % yield). ^1^H NMR (400 MHz, CDCl_3_): *δ* (ppm)=13.26 (bs, 1H), 9.08 (bs, 1H), 8.53 (d, 1H), 7.94 (d, 2H), 7.66 (t, 1H), 7.58 (dd, 2H), 6.14 (d, 1H), 5.88 (m, 1H), 5.47 (m, 1H), 4.43 (t, 2H), 4.31 (t, 2H), 3.23 (s, 3H), 1.80 (s, 3H). ^13^C NMR (100 MHz, CDCl_3_): *δ* (ppm)=176.68, 167.05, 166.88, 146.88, 144.97, 136.72, 135.79, 133.76, 131.46, 129.19, 127.56, 125.88, 125.19, 113.96, 104.64, 62.58, 52.84, 40.07, 18.13. HRMS (ESI+): *m*/*z* calculated for C_21_H_22_N_5_O_4_S ([M+H])^+^ 440.1392, found ([M+H])^+^ 440.1441. UPLC: *t*_R_=4.41 min (100 % peak area).

### Preparation of fluorescent MIP particles

**2** **c** (0.66 mg, 0.0015 mmol), ENOX‐TBA (0.84 mg, 0.0015 mmol), HEMA (6.95 μL, 0.056 mmol) and EGDMA (15.73 μL, 0.083 mmol) were dissolved in 1.75 ml ACN using an ultrasonic bath for 10 min. Afterwards, 10 mg of RAFT agent‐functionalized SiO_2_ particles were added and sonicated for another 10 min. Later, 2,2′‐azobis(2,4‐dimethyl) valeronitrile (ABDV, 0.6 mg, 0.0024 mmol) was added under cooling with ice and the solution was flushed with Argon for 5 min. The mixture was polymerized for 18 h at 50 °C and then for 2 h at 70 °C. After adding 30 ml hexane, the synthesized particles were washed once with 15 ml CHCl_3_ and twice with 15 ml ACN with centrifugation at 9000 rpm for 5 min in between. For the removal of template, a MeOH/acetic acid 99/1 (v/v) mixture was used. Subsequently, the particles were washed again with ACN three times (centrifugation at 9000 rpm for 5 min) and left in the vacuum oven overnight for drying. Repetitive measurements of the particles over time revealed that they are stable in dry form for at least 30 months.

### Spectroscopic details

For the measurement of the pre‐polymerization mixture, micro‐cells with a path length of 100 μm were used. The fluorescence quantum yields (*Φ*_f_) of **2** **b** and **2** **c** were determined relative to ruthenium(II) tris(bipyridine) dichloride, ([Ru(bpy)_3_]Cl_2_ (bpy: 2,2′‐bipyridine) in aerated water *Φ*_f_=0.040.^[38]^ The molar absorption coefficient of **2** **c** was determined to *ϵ*_464_=7836±196 cm^−1^ M^−1^ from triplicate measurements of three separately weighted samples (*N*=9).

## Conflict of interest

The authors declare no conflict of interest.

1

## Supporting information

As a service to our authors and readers, this journal provides supporting information supplied by the authors. Such materials are peer reviewed and may be re‐organized for online delivery, but are not copy‐edited or typeset. Technical support issues arising from supporting information (other than missing files) should be addressed to the authors.

Supporting Information

## Data Availability

The data that support the findings of this study are available from the corresponding author upon reasonable request.
