# Supplementary material for: Thiourea‐ and Amino‐Substituted Benzoxadiazole Dyes with Large Stokes Shifts as Red‐Emitting Probe Monomers for Imprinted Polymer Layers Targeting Carboxylate‐Containing Antibiotics
Source: Chemistry. 2022 Mar 15;28(21):e202104525. doi: 10.1002/chem.202104525 (PMC9310751; doi:10.1002/chem.202104525)
Supplement: Supplementary file 1 — Supporting Information [file CHEM-28-0-s001.pdf]

Chemistry–A European Journal

Supporting Information

Thiourea- and Amino-Substituted Benzoxadiazole Dyes with Large Stokes Shifts as Red-Emitting Probe Monomers for Imprinted Polymer Layers Targeting Carboxylate-Containing Antibiotics

Virginia Valderrey, Kornelia Gawlitza, and Knut Rurack*

Supporting Information

Contents

1. EXPERIMENTAL DETAILS **S2**

1.1 GENERAL INFORMATION AND INSTRUMENTATION S2

1.2 BENZOXADIAZOLE SYNTHESES S4

1.3 NMR SPECTRA OF THIOUREA-SUBSTITUTED BENZOXADIAZOLE PROBES S8

2. SPECTROSCOPIC DATA **S13**

3. MOLECULARLY IMPRINTED POLYMER (MIP) SYNTHESIS **S17**

4. PARTICLE CHARACTERIZATION: ZETA POTENTIAL AND THERMOGRAVIMETRIC ANALYSIS (TGA) **S18**

5. FLUORESCENCE MEASUREMENTS WITH MIP SENSOR PARTICLES IN ACN **S18**

6. TEM IMAGES **S19**

7. ABSORPTION SPECTRA OF 2C AND 2C-CONTAINING ENOX-TBA-IMPRINTED CORE-SHELL SILICA PARTICLES IN ACN. **S19**

8. FLUORESCENCE SPECTRA OF MIP PARTICLES WITH AMOX-TBA AND AMPI-TBA **S20**

9. UNCERTAINTY BUDGET **S21**

10. REFERENCES **S21**

1. Experimental details

1.1 General information and instrumentation

Chemicals were purchased from commercial suppliers and used without further purification unless it is specifically indicated. 4-Chloro-7-nitro-2,1,3-benzoxadiazole (99%), 2-aminoethyl methacrylate hydrochloride (90%) and iodomethane (99%) were obtained from Sigma-Aldrich; sodium hydroxide (≥99.0%) from Merck; triethylamine (99.50%) from Applichem; and iron powder (99%) from Alfa Aesar; benzoyl isothiocyanate (98%) from Chempur; 4-nitrophenyl isothiocyanate (98%) from Acros. Tetrabutylammonium hydroxide (TBA-OH) 1 M methanol solution, tetraethyl orthosilicate (TEOS), 32 % ammonia solution in water, (3-aminopropyl)triethoxysilane (APTES), 2-hydroxyethyl methacrylate (HEMA), ethylene glycol di-methacrylate (EGDMA), ampicillin (AMPI) (96%), amoxicillin (AMOX) (90%) and aluminum oxide type 5016A basic (particle size 50–200 µm) were obtained from Sigma Aldrich/Merck, benzoyl isothiocyanate (98%) from Chempur. Triethylamine (TEA) and 37 % hydrochloric acid were obtained from AppliChem. 4-Cyano-4-(phenylcarbonothioylthio)pentanoic acid (CPDB) was purchased from abcr. Ethyl chloroformate (ECF) was purchased from Fluka and 2,2′-azobis(2,4-dimethylvaleronitril) (ABDV) initiator from Wako Chemicals. Enoxacin (ENOX) and aminomethylphosphonic acid (AMPA) were obtained from Alfa Aesar. All organic solvents used were of HPLC grade quality for organic and particle synthesis and spectroscopic grade for UV/Vis and fluorescence titrations and used as received from the supplier. Acetic acid glacial (≥99.0%), acetonitrile (≥99.95%), acetone (≥99.0%), dichloromethane (≥99.9%), ethanol 96% (≥95.1%), ethanol absolute (≥99.9%), ethyl acetate (≥99.5%), hydrochloric acid (37%) and *N*,*N*-dimethylformamide (≥99.9%). Milli-Q® water was drawn from a Milli-Q® ultrapure water purification system (Millipore Synthesis A10). For column chromatography, silica gel (0.035-0.070 mm, 60 Å) was used.

NMR spectra were recorded at a Varian Mercury 400 NMR spectrometer. Samples were dissolved in deuterated solvent ($CDCl_3$), using residual proton signals as standard (^1H: δ($CHCl_3$ = 7.26 ppm), (^{13}C: δ($CHCl_3$ = 77.16 ppm). Chemical shifts are represented in δ (ppm). Ultra-high performance liquid chromatography electro-spray ionization mass spectrometry (UPLC-ESI-MS) was performed on a Waters Acquity UPLC (gradient mixtures of acetonitrile/water) with a Waters LCT Premier XE mass detector. Additionally, a Waters Alliance System with Waters Separations Module 2695, a Waters Diode Array Detector 996 and a Waters Mass Detector ZQ 2000 were used. Chromatographic separations were performed with a gradient acetonitrile in water with 0.1% formic acid. Absorption spectra and spectrophotometric titrations were acquired with a Specord 210 Plus spectrometer (Analytik Jena). Fluorescence spectra and titrations were carried out on a FluoroMax 4 spectrofluorometer (Horiba Jobin-Yvon). Absorption spectra and spectrophotometric titrations were acquired using diluted solutions with an absorbance of ca. 0.1 at the longest wavelength absorption maximum. UV/Vis and fluorescence titrations were performed by adding solutions containing the analyte (AcO-TBA or ENOX-TBA) to solutions of the benzoxadiazole dyes in a 1cm path cell in the indicated solvent. In all cases the corresponding dye solution was employed also as the analyte solution in order to avoid dilution effects. The binding constants were assessed with the software BindFit v0.5.[1] The uncertainties of the fluorescence quantum yields were determined to ±10% (for Φ_f > 0.02) and ±20% (for 0.02 > Φ_f). For the calculation of the molar absorption coefficient the dilution was chosen in such a way that the absorbances of the sample solutions equalled 0.15 ± 0.02 at the maximum of the first absorption band. Transmission electron microscopy (TEM) was conducted with a FEI Talos™ F200S

operating at 200 kV. Dynamic light scattering experiments were performed with a Zetasizer Nano ZS (Malvern Instruments). Samples for the TEM measurements were prepared on carbon thin film-modified copper grids (200 mesh) by drying 10 µL of a 0.1% (w/v) dispersion in acetonitrile. ImageJ software[2] (National Institute of Health, US) was used for calculating the diameter and the shell thickness of the used particles by collecting the data from 40 particles and calculating the average and standard deviation of the measurements. For zeta potential measurements, 0.04 mg mL^{-1} particle suspensions were prepared in Milli-Q water and measurements performed using disposable folded capillary cells.

1.2 Benzoxadiazole syntheses

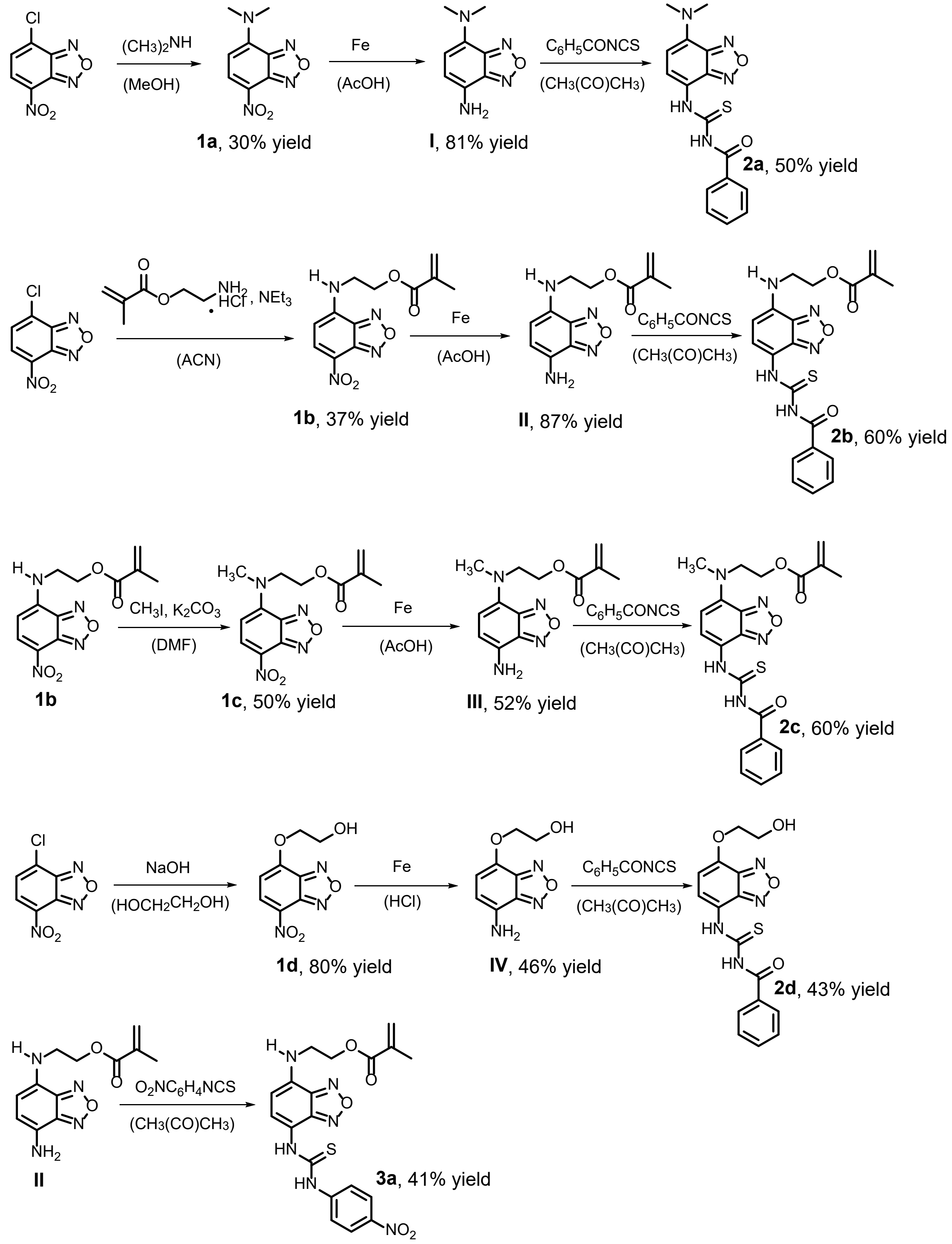

Scheme S1. Synthetic scheme for the preparation of thiourea-substituted bezoxadiazole probes and its synthetic intermediates.

***N,N*-Dimethyl-7-nitrobenzo[c][1,2,5]oxadiazol-4-amine** (**1a**) was synthesized following described procedures[3]
^{1}H-NMR (400 MHz, $CDCl_3$): δ (ppm) = 8.44 (d, 1H), 6.08 (d, 1H), 3.63 (s, 3H).

2-((7-Nitrobenzo[c][1,2,5]oxadiazol-4-yl)amino)ethyl methacrylate (**1b**).
Chloro-7-nitro-2,1,3-benzoxadiazole (99%) (0.60 g, 3.00 mmol) and 2-aminoethyl methacrylate hydrochloride (90%) (0.50 g, 3.00 mmol) were suspended in 75 ml of acetonitrile (ACN). Afterwards, triethylamine (99.5%) (0.63 ml, 4.50 mmol) was dropwise added and the reaction mixture was stirred overnight at room temperature. The reaction mixture was concentrated and purified by silica column chromatography with DCM as mobile phase obtaining the desired compound (0.32 g, 37% yield) as a yellow solid.
^{1}H-NMR (400 MHz, $CDCl_3$): δ (ppm) = 8.48 (d, 1H), 6.63 (bs, 1H), 6.15 (m, 1H), 5.64 (m, 1H), 4.54 (t, 2H), 3.84 (q, 2H), 1.95 (s, 3H). ^{13}C-NMR (100 MHz, $CDCl_3$): δ (ppm) = 167.53, 144.27, 143.76, 143.51, 136.05, 135.43, 127.04, 98.93, 61.97, 43.26, 29.66, 18.23. HRMS (ESI^-): m/z calculated for $C_{12}H_{12}N_4O_5$ ($[M-H]^-$) 291.0729, found ($[M-H]^-$) 291.0744. UPLC: t_R = 3.99 min (100 % peak area).

2-(Methyl(7-nitrobenzo[c][1,2,5]oxadiazol-4-yl)amino)ethyl methacrylate (**1c**).
2-((7-Nitrobenzo[c][1,2,5]oxadiazol-4-yl)amino)ethyl methacrylate (**1b**) (0.10 g, 0.35 mmol) was dissolved in 2.0 ml DMF, followed by the addition of K_2CO_3 (0.1 g, 0.52 mmol). Iodomethane (99%) (0.04 ml, 0.70 mmol) was afterwards added and the reaction mixture was stirred for 4 hours. Later, it was diluted with EtOAc (25 ml) and extracted with water (3 × 25 ml) and one time with Brine (20 ml). The organic phase was dried over $MgSO_4$ and concentrated under vacuum to obtain a red oil (0.05 g, 50% yield).
^{1}H-NMR (400 MHz, $CDCl_3$): δ (ppm) = 8.46 (d, 1H), 6.18 (bs, 1H), 5.92 (m, 1H), 5.52 (m, 1H), 4.53-4.50 (m, 4H), 3.48 (s, 3H), 1.82 (s, 3H). ^{13}C-NMR (100 MHz, $CDCl_3$): δ (ppm) = 166.82, 145.28, 144.61, 144.52, 135.49, 135.09, 126.39, 101.76, 61.99, 54.19, 42.04, 18.23. HRMS (ESI+): m/z calculated for $C_{13}H_{14}N_4O_5$ ($[M+H]^+$) 307.1042, found ($[M+H]^+$) 307.1077.UPLC: t_R = 3.06 min (100 % peak area).

***N*^{4},*N*^{4}-Dimethylbenzo[c][1,2,5]oxadiazole-4,7-diamine** (**I**).[3]
N,*N*-Dimethyl-7-nitrobenzo[c][1,2,5]oxadiazol-4-amine (**1a**) (0.208 g, 1.00 mmol) was suspended in 3.0 ml of acetic acid. Afterwards, iron powder (99.0%) (0.167 g, 3.0 mmol) was added and the reaction mixture was stirred for 1 h at room temperature. The reaction mixture was diluted with 20 ml of EtOAc and the organic phase was extracted with saturated $NaHCO_3$ (3 × 20 ml) and one time with 20 ml of Brine. The organic phase was collected, dried over $MgSO_4$ and concentrated in a vacuum. The reaction mixture was purified by silica column chromatography with EtOAc as mobile phase to obtain the desired compound as a red solid (0.144 g, 81% yield).
^{1}H-NMR (400 MHz, $CDCl_3$): δ (ppm) = 6.31 (d, 1H), 6.11 (d, 1H), 4.00 (bs, 2H), 3.08 (s, 6H).

2-((7-Aminobenzo[c][1,2,5]oxadiazol-4-yl)amino)ethyl methacrylate (**II**).
2-((7-Nitrobenzo[c][1,2,5]oxadiazol-4-yl)amino)ethyl methacrylate (**1b**) (0.132 g, 0.45 mmol) was suspended in 4.0 ml of acetic acid. Afterwards, iron powder (99.0%) (0.45 g, 8.10 mmol) was added and the reaction mixture was stirred for one hour at room temperature. The reaction mixture was diluted with 20 ml of EtOAc and the organic phase was extracted with saturated $NaHCO_3$ (3 × 20 ml) and one time with 20 ml of brine. The organic phase was collected, dried over $MgSO_4$ and concentrated in a vacuum. The reaction mixture was purified by silica column chromatography with EtOAc as mobile phase to obtain the desired compound as a red solid (0.10 g, 87% yield).

^{1}H-NMR (400 MHz, $CDCl_3$): δ (ppm) = 6.32 (d, 1H), 6.13 (m, 1H), 6.07 (d, 1H), 5.59 (m, 1H), 4.42 (m, 2H), 3.55 (m, 2H), 1.95 (s, 3H). ^{13}C-NMR (100 MHz, $CDCl_3$): δ (ppm) = 167.53, 145.47, 144.26, 143.48, 135.89, 127.86, 126.20, 110.57, 104.75, 62.80, 42.84, 18.23. HRMS (ESI+): m/z calculated for $C_{12}H_{14}N_4O_3$ ([M+H])$^+$ 263.1144, found ([M+H])$^+$ 263.1155. UPLC: t_R = 2.19 min (100 % peak area).

2-((7-Aminobenzo[c][1,2,5]oxadiazol-4-yl)(methyl)amino)ethyl methacrylate (**III**).
2-(Methyl(7-nitrobenzo[c][1,2,5]oxadiazol-4-yl)amino)ethyl methacrylate (**1c**) (0.100 g, 0.32 mmol) was suspended in 2.0 ml of acetic acid. Afterwards, iron powder (99.0%) (0.328 g, 5.87 mmol) was added and the reaction mixture was stirred for one hour at room temperature. The reaction mixture was diluted with 100 ml of EtOAc and the organic phase was extracted with saturated $NaHCO_3$ (3 × 100 ml). The organic phase was collected, dried over $MgSO_4$ and concentrated in a vacuum. The reaction mixture was purified by silica column chromatography with EtOAc as mobile phase to obtain the desired compound as a red solid (0.77 g, 52% yield).
^{1}H-NMR (400 MHz, $CDCl_3$): δ (ppm) = 6.31 (d, 1H), 6.08 (d, 1H), 5.86 (m, 1H), 5.44 (m, 1H), 4.34 (t, 2H), 4.07 (t, 2H), 3.99 (bs, 2H), 3.05 (s, 3H), 1.79 (m, 3H). ^{13}C-NMR (100 MHz, $CDCl_3$): δ (ppm) = 167.14, 145.94, 145.80, 135.96, 130.88, 125.54, 110.12, 109.76, 62.49, 52.56, 39.58, 18.09. HRMS (ESI+): m/z calculated for $C_{13}H_{16}N_4O_3$ ([M+H])$^+$ 277.1303, found ([M+H])$^+$ 277.1349.
UPLC: t_R = 2.19 min (100 % peak area).

2-((7-(3-Benzoylthioureido)benzo[c][1,2,5]oxadiazol-4-yl)(dimethyl) (**2a**).
N,*N*-Dimethyl-7-nitrobenzo[c][1,2,5]oxadiazol-4-amine (**1a**) (0.145 g, 0.70 mmol) was dissolved in 4.0 ml of acetone. Benzoyl isothiocyanate (98.0%) (0.095 ml, 0.70 mmol) was added and an orange precipitate was formed after 5 min. The precipitate was filtered to afford a red solid as the desired compound (0.11 g, 50 % yield).
^{1}H-NMR (400 MHz, $CDCl_3$): δ (ppm) = 13.18 (bs, 1H), 9.11 (bs, 1H), 8.49 (d, 1H), 7.94 (d, 2H), 7.66 (t, 1H), 7.56 (t, 2H), 6.09 (d, 1H), 3.34 (s, 6H). ^{13}C-NMR (100 MHz, $CDCl_3$): δ (ppm) = 176.74, 166.88, 146.97, 145.42, 138.09, 133.75, 131.55, 129.22, 127.58, 125.44, 113.54, 104.17, 42.05. HRMS (ESI+): m/z calculated for $C_{16}H_{15}N_5O_2S$ ([M+H])$^+$ 342.1025, found ([M+H])$^+$ 342.1072. UPLC: t_R = 5.03 min (100 % peak area).

2-((7-(3-Benzoylthioureido)benzo[c][1,2,5]oxadiazol-4-yl)amino)ethyl methacrylate (**2b**).
2-((7-Aminobenzo[c][1,2,5]oxadiazol-4-yl)amino)ethyl methacrylate (**II**) (0.102 g, 0.39 mmol) was dissolved in 5.0 ml of acetone. Benzoyl isothiocyanate (98.0%) (0.056 ml, 0.39 mmol) were added and an orange precipitate was formed after 5 min. The precipitate was filtered to afford an orange solid as the desired compound (0.1 g, 60 % yield).
^{1}H-NMR (400 MHz, DMSO-d6): δ (ppm) = 12.84 (bs, 1H), 11.71 (bs, 1H), 8.02 (d, 1H), 7.99 (m, 2H), 7.66 (t, 1H), 7.55 (t, 2H), 7.36 (t, 1H), 6.32 (d, 1H), 6.02 (m, 1H), 5.65 (m, 1H), 5.27 (m, 1H), 4.33 (m, 2H), 3.62 (m, 2H), 1.86 (s, 3H). ^{13}C-NMR (100 MHz, DMSO-d6): δ (ppm) = 179.80, 169.01, 167.06, 147.02, 145.24, 136.19, 135.53, 133.63, 132.47, 129.58, 129.17, 128.89, 126.45, 112.84, 101.04, 62.97, 42.12, 18.44. HRMS (ESI+): m/z calculated for $C_{20}H_{19}N_5O_4S$ ([M+H])$^+$ 426.1236, found ([M+H])$^+$ 426.1236. UPLC: t_R = 4.16 min (100 % peak area).

2-((7-(3-Benzoylthioureido)benzo[c][1,2,5]oxadiazol-4-yl)(methyl)amino)ethyl methacrylate (**2c**).
2-((7-Aminobenzo[c][1,2,5]oxadiazol-4-yl)(methyl)amino)ethyl methacrylate (**III**) (0.098 g, 0.35 mmol) was dissolved in 3.0 ml of acetone. Benzoyl isothiocyanate (98.0%) (0.048 ml, 0.35 mmol)

was added and an orange precipitate was formed after 5 min. The precipitate was filtered to afford an orange solid as the desired compound (0.090 g, 60 % yield).
^{1}H-NMR (400 MHz, $CDCl_3$): δ (ppm) = 13.26 (bs, 1H), 9.08 (bs, 1H), 8.53 (d, 1H), 7.94 (d, 2H), 7.66 (t, 1H), 7.58 (dd, 2H), 6.14 (d, 1H), 5.88 (m, 1H), 5.47 (m, 1H), 4.43 (t, 2H), 4.31 (t, 2H), 3.23 (s, 3H), 1.80 (s, 3H). ^{13}C-NMR (100 MHz, $CDCl_3$): δ (ppm) = 176.68, 167.05, 166.88, 146.88, 144.97, 136.72, 135.79, 133.76, 131.46, 129.19, 127.56, 125.88, 125.19, 113.96, 104.64, 62.58, 52.84, 40.07, 18.13. HRMS (ESI+): m/z calculated for $C_{21}H_{22}N_5O_4S$ ([M+H])$^+$ 440.1392, found ([M+H])$^+$ 440.1441. UPLC: t_R = 4.41 min (100 % peak area).

2-((7-Nitrobenzo[c][1,2,5]oxadiazol-4-yl)oxy)ethanol (**1d**) was synthesized according to ref[4]
^{1}H-NMR (400 MHz, $CDCl_3$): δ (ppm) = 8.54 (d, 1H), 6.74 (d, 1H), 4.51 (t, 2H), 4.17 (t, 2H), 2.21 (bs, 1H). ^{13}C-NMR (100 MHz, $CDCl_3$): δ (ppm) = 154.48, 145.16, 143.95, 133.82, 104.90, 72.34, 60.52. HRMS (ESI-): m/z calculated for $C_8H_6N_3O_5$ ([M-H])$^-$ 224.0307, found ([M-H])$^-$ 224.0281. UPLC: t_R = 1.20 min (100 % peak area).

2-((7-Aminobenzo[c][1,2,5]oxadiazol-4-yl)oxy)ethanol (**IV**).
2-(7-Nitrobenzo[c][1,2,5]oxadiazol-4-yloxy)ethanol (**1d**) (0.098 g, 0.44 mmol) was suspended in 2.5 ml of concentrated HCl. Later, iron powder (99.0%) (0.273 g, 4.88 mmol) was added and the reaction mixture was stirred for one hour at room temperature. A NaOH (2M) aqueous solution was added until the pH = 7. The reaction mixture was extracted with DCM (5 x 40 ml) and the organic phase was concentrated under vacuum to obtain the desired compound as a brown solid (0.04 g, 46% yield) which was used in the next reaction step without further purification.
^{1}H-NMR (400 MHz, $CDCl_3$): δ (ppm) = 6.47 (d, 1H), 6.26 (d, 1H), 4.23 (t, 2H), 4.03 (t, 2H). HRMS (ESI+): m/z calculated for $C_8H_{10}N_3O_3$ ([M+H])$^+$ 196.0722, found ([M+H])$^+$ 196.0720. UPLC: t_R = 2.78 min (100 % peak area).

***N*-((7-(2-Hydroxyethoxy)benzo[c][1,2,5]oxadiazol-4-yl)carbamothioyl)benzamide** (**2d**).
2-(7-Aminobenzo[c][1,2,5]oxadiazol-4-yloxy)ethanol (**IV**) (0.025 g, 0.13 mmol) was dissolved in 1.6 ml of acetone. Benzoyl isothiocyanate (98.0%) (0.018 ml, 0.13 mmol) was added and a yellow precipitate was after formed. The precipitate was filtered to afford a yellow solid as the desired compound (0.020 g, 43 % yield).
^{1}H-NMR (400 MHz, $CDCl_3$): δ (ppm) = 13.49 (bs, 1H), 9.13 (bs, 1H), 8.75 (d, 1H), 7.94 (d, 2H), 7.68 (t, 1H), 7.57 (t, 2H), 6.66 (d, 1H), 4.36 (t, 2H), 4.10 (t, 2H). ^{13}C-NMR (100 MHz, $CDCl_3$): δ (ppm) = 183.29, 177.22, 167.02, 146.85, 145.23, 144.82, 134.07, 131.24, 129.20, 127.26, 122.37, 119.33, 109.71, 107.36, 71.03, 60.98. HRMS (ESI+): m/z calculated for $C_{16}H_{15}N_4O_4S$ ([M+H])$^+$ 359.0814, found ([M+H])$^+$ 359.0786. UPLC: t_R = 4.06 min (100 % peak area).

2-((7-(3-(4-Nitrophenyl)thioureido)benzo[c][1,2,5]oxadiazol-4-yl)amino)ethyl methacrylate (**3a**).
2-((7-Aminobenzo[c][1,2,5]oxadiazol-4-yl)amino)ethyl methacrylate (**II**) (0.079 g, 0.22 mmol) was dissolved in 4.0 ml of acetone. 4-Nitrophenyl isothiocyanate (98.0%) (0.040 ml, 0.22 mmol) were added and the reaction mixture was stirred at room temperature overnight. An orange precipitate was formed and filtered to afford an orange solid as the desired compound (0.040 g, 41 % yield).
^{1}H-NMR (400 MHz, DMSO-d6): δ (ppm) = 10.2 (bs, 1H), 10.13.00 (bs, 1H), 8.18 (d, 2H), 7.85 (d, 1H), 7.32 (d, 1H), 6.29 (d, 1H), 6.03 (m, 1H), 5.65 (m, 1H), 4.32 (m, 2H), 3.60 (m, 2H), 1.85 (s, 3H). ^{13}C-NMR (100 MHz, DMSO-d6): δ (ppm) = 180.66, 167.07, 147.91, 146.68, 145.72, 143.06, 136.19,

135.83, 132.09, 126.46, 124.57, 122.79, 112.78, 101,19, 62.88, 42.09, 18.43. HRMS (ESI+): m/z calculated for $C_{19}H_{19}N_6O_5S$ ($[M+H]^+$) 443.1138, found ($[M+H]^+$) 443.1156. UPLC: t_R = 4.43 min (100 % peak area).

1.3 NMR spectra of thiourea-substituted benzoxadiazole probes

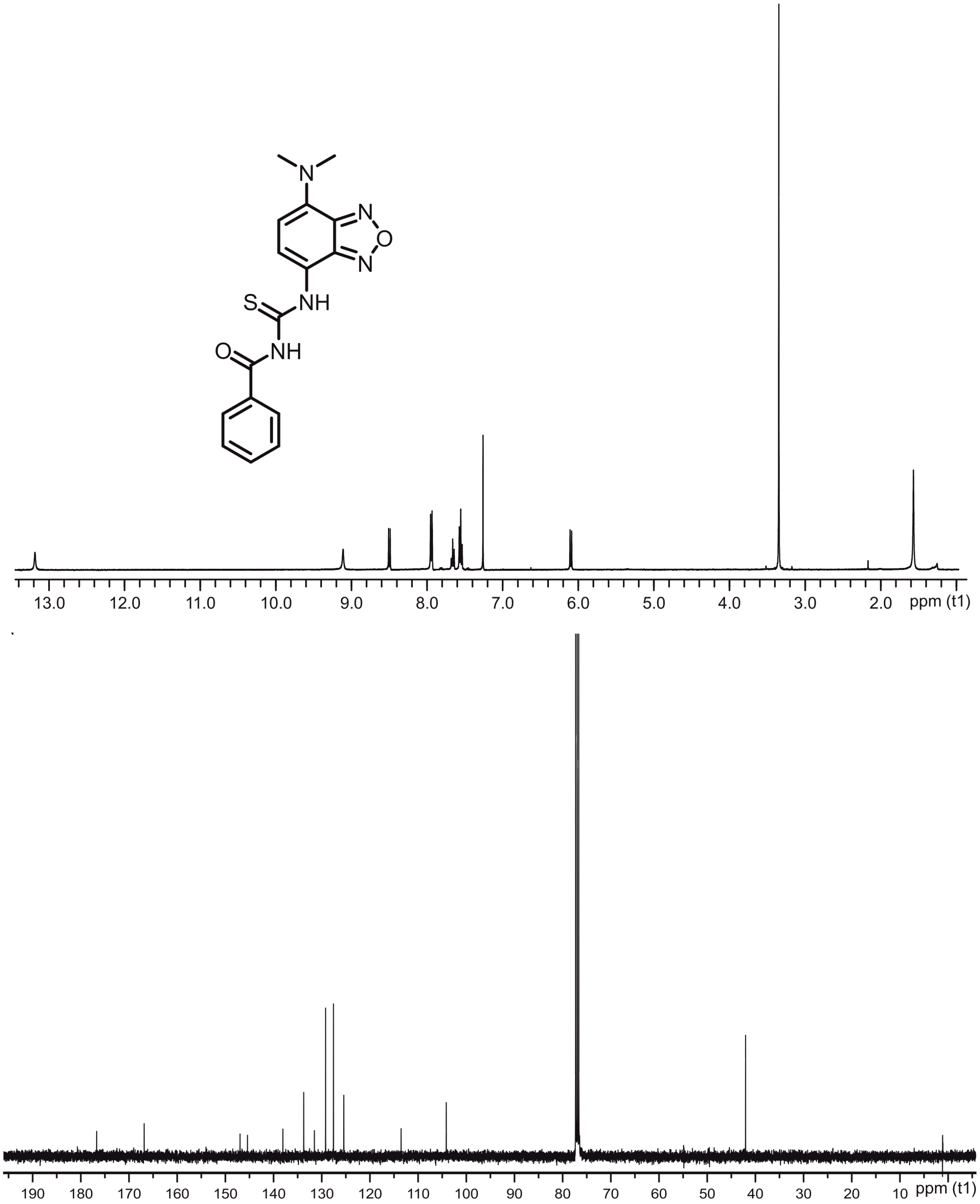

Figure S1. ^1H-NMR (400 MHz, $CDCl_3$) (top) and ^{13}C-NMR (100 MHz, $CDCl_3$) (bottom) spectra of **2a**.

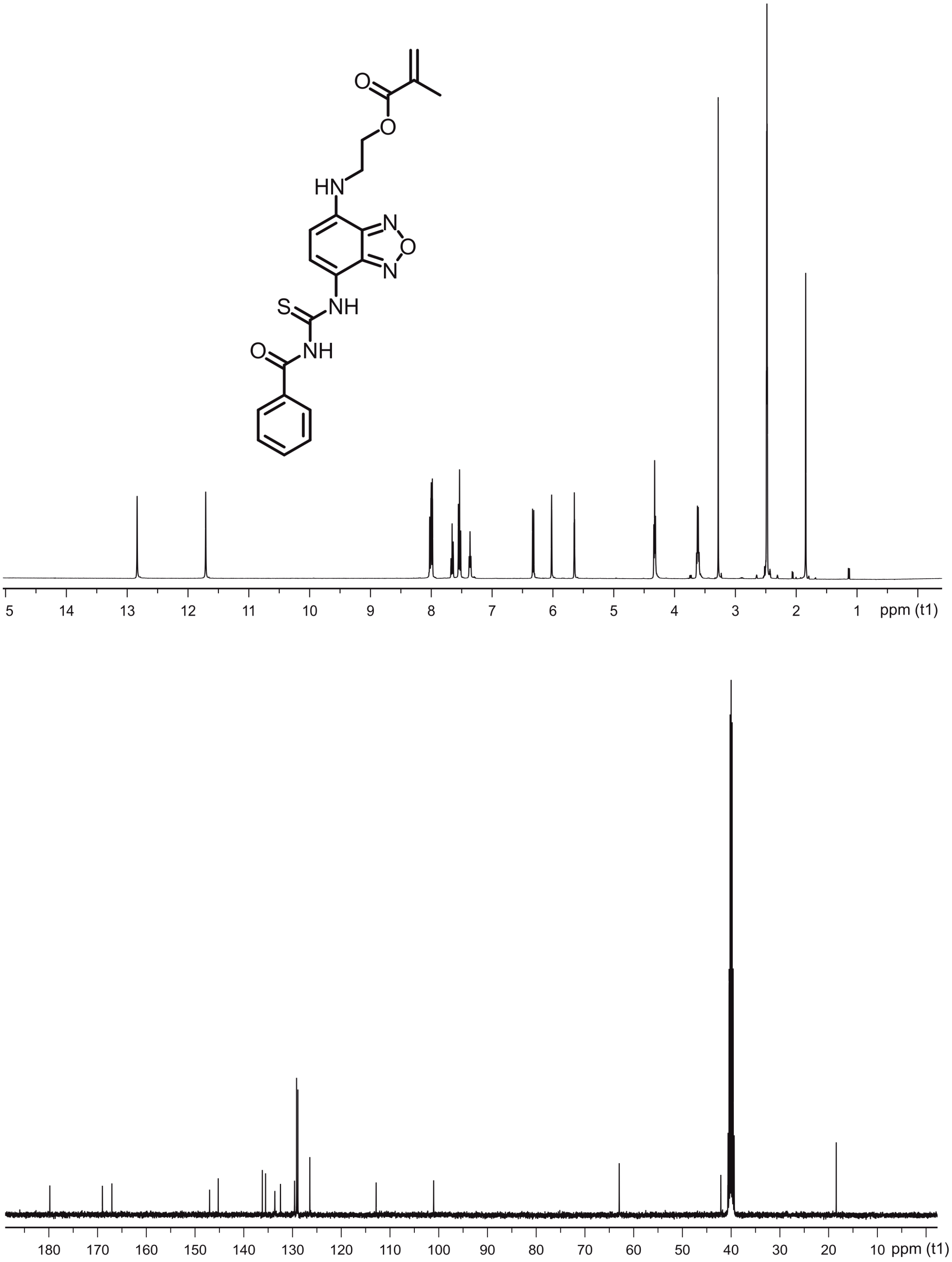

Figure S2. ^{1}H-NMR (400 MHz, DMSO-d6) (top) and ^{13}C-NMR (100 MHz, DMSO-d6) (bottom spectra of **2b**.

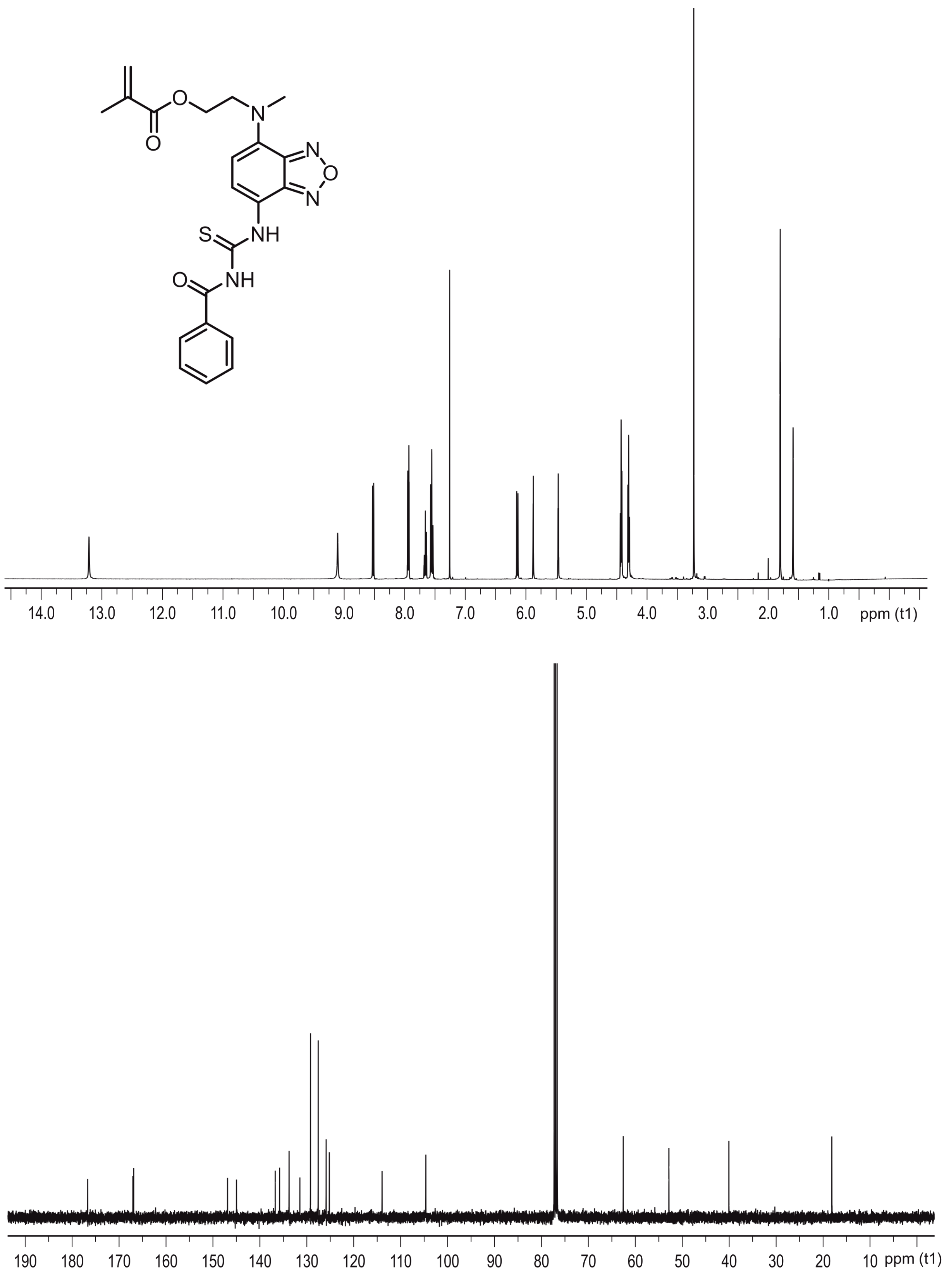

Figure S3. ^{1}H-NMR (400 MHz, $CDCl_3$) (top) and ^{13}C-NMR (100 MHz, $CDCl_3$) (bottom) spectra of **2c**.

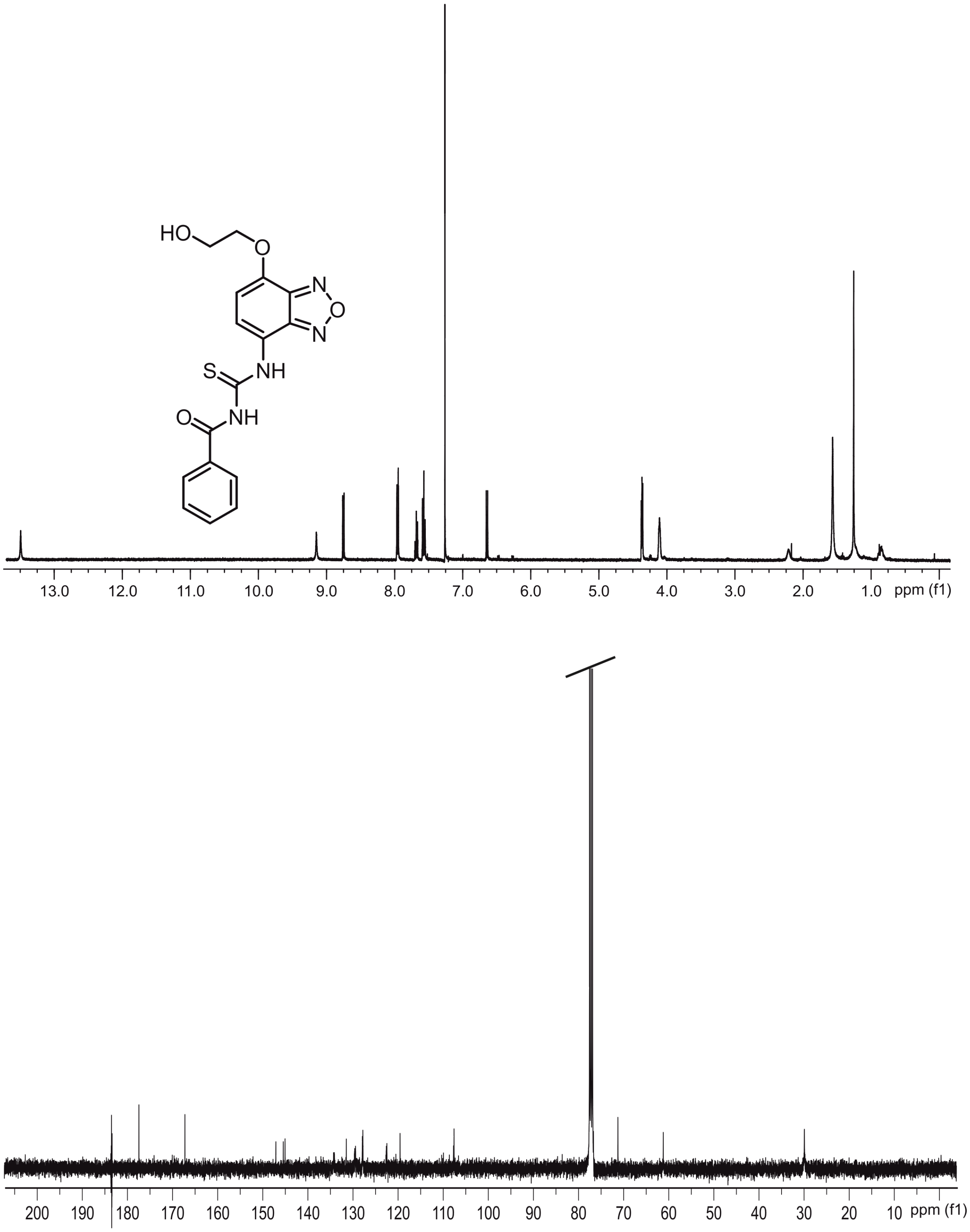

Figure S4. ^{1}H-NMR (400 MHz, $CDCl_3$) (top) and ^{13}C-NMR (100 MHz, $CDCl_3$) (bottom) spectra of **2d**.

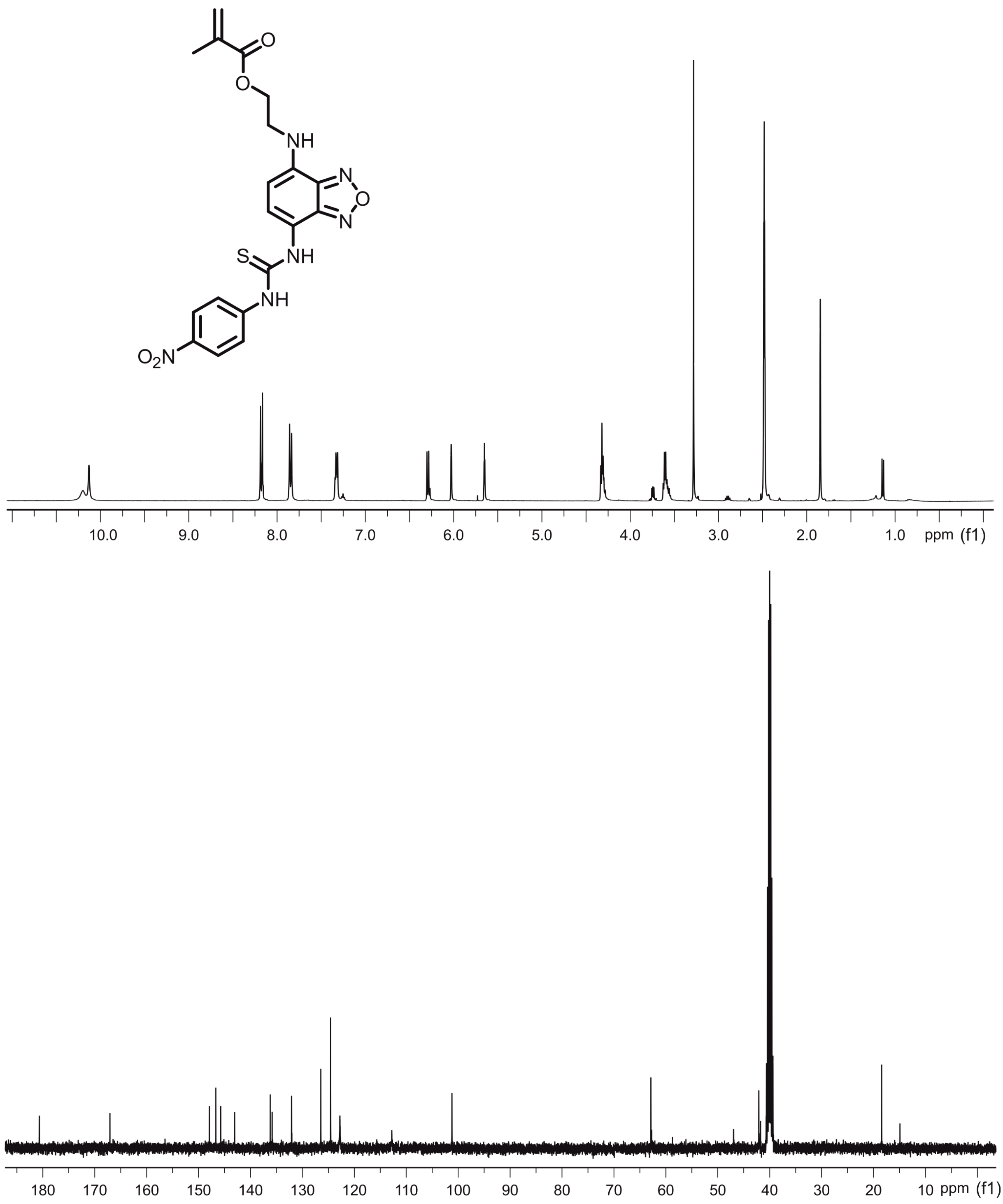

Figure S5. ^{1}H-NMR (400 MHz, DMSO-d6) (top) and ^{13}C-NMR (100 MHz, DMSO-d6) spectrum of **3a**.

2. Spectroscopic data

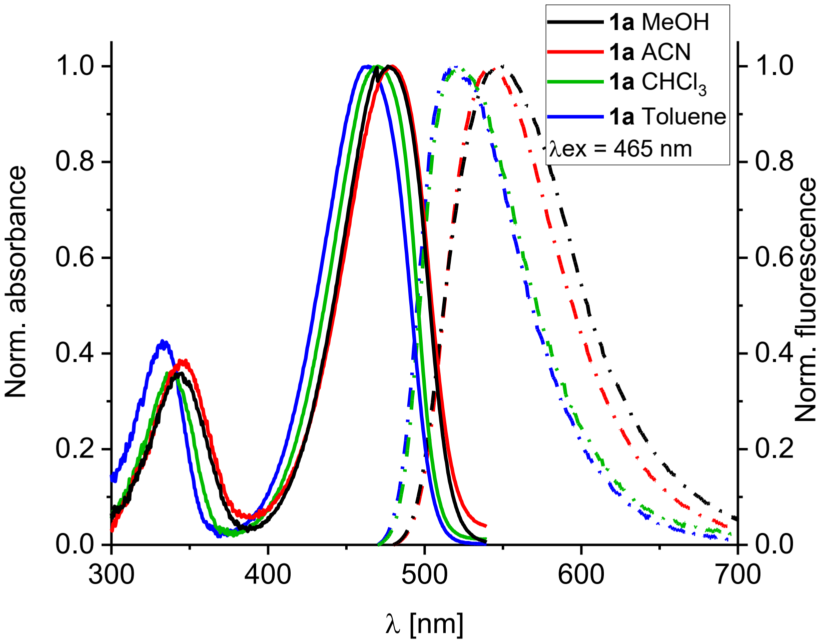

Figure S6. UV/Vis absorption and emission spectra of **1a** in solvents of different polarity.

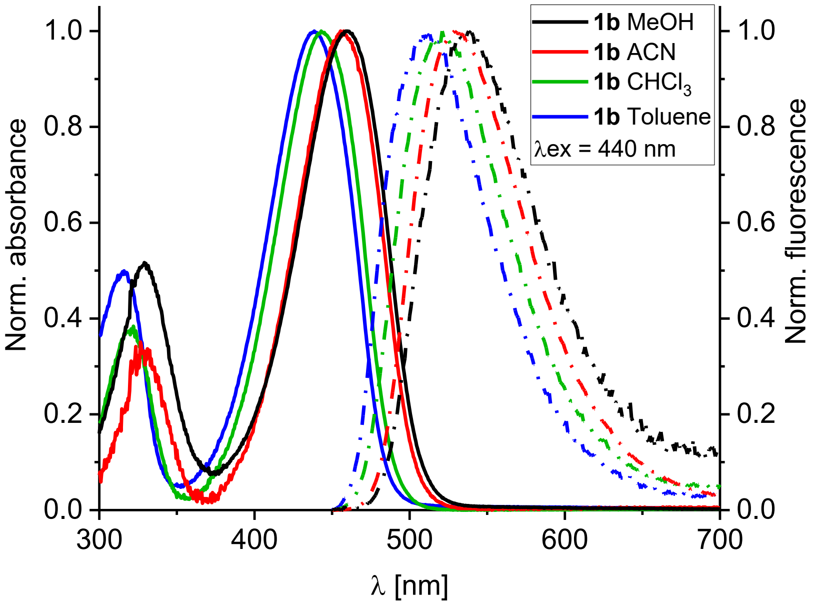

Figure S7. UV/Vis absorption and emission spectra of **1b** in solvents of different polarity.

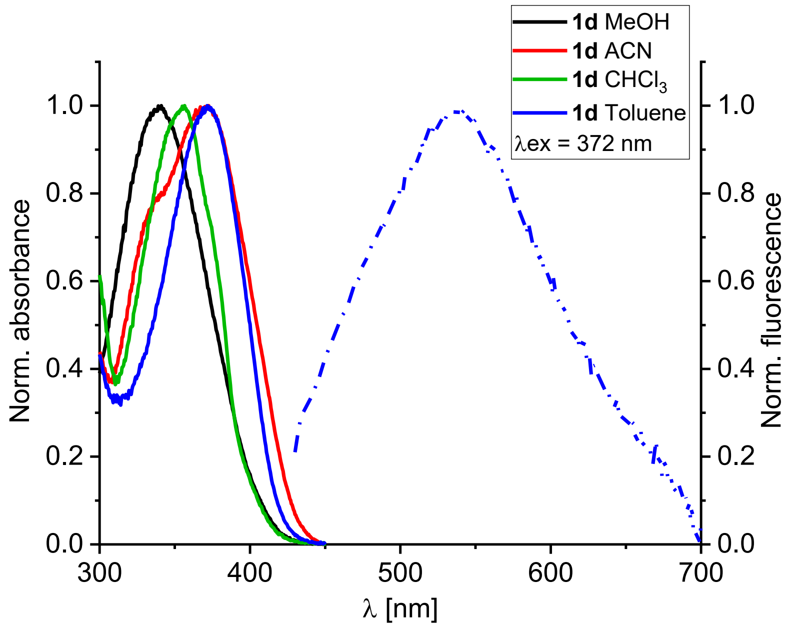

Figure S8. UV/Vis absorption and emission spectra of **1d** in solvents of different polarity. No fluorescence emission was observed for **1d** in $CHCl_3$, ACN and MeOH.

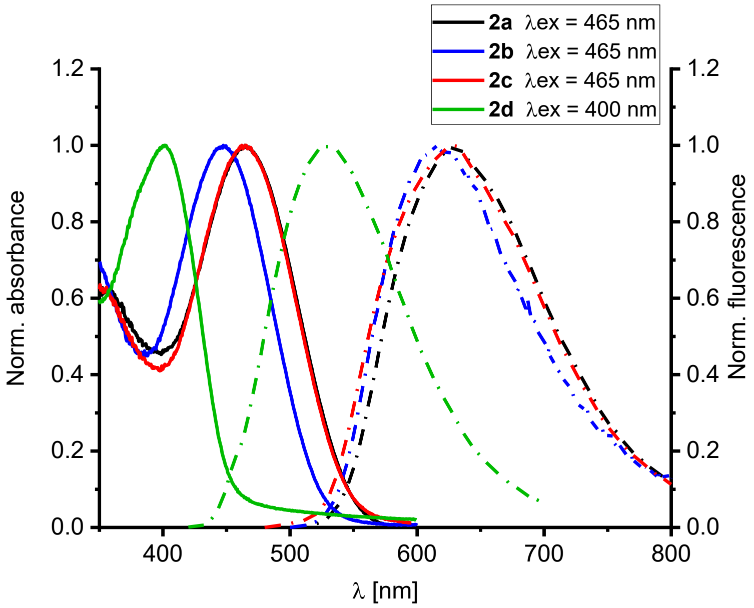

Figure S9. UV/Vis absorption and emission spectra of the thiourea substituted benzoxadiazoles series **2a–2d** in toluene.

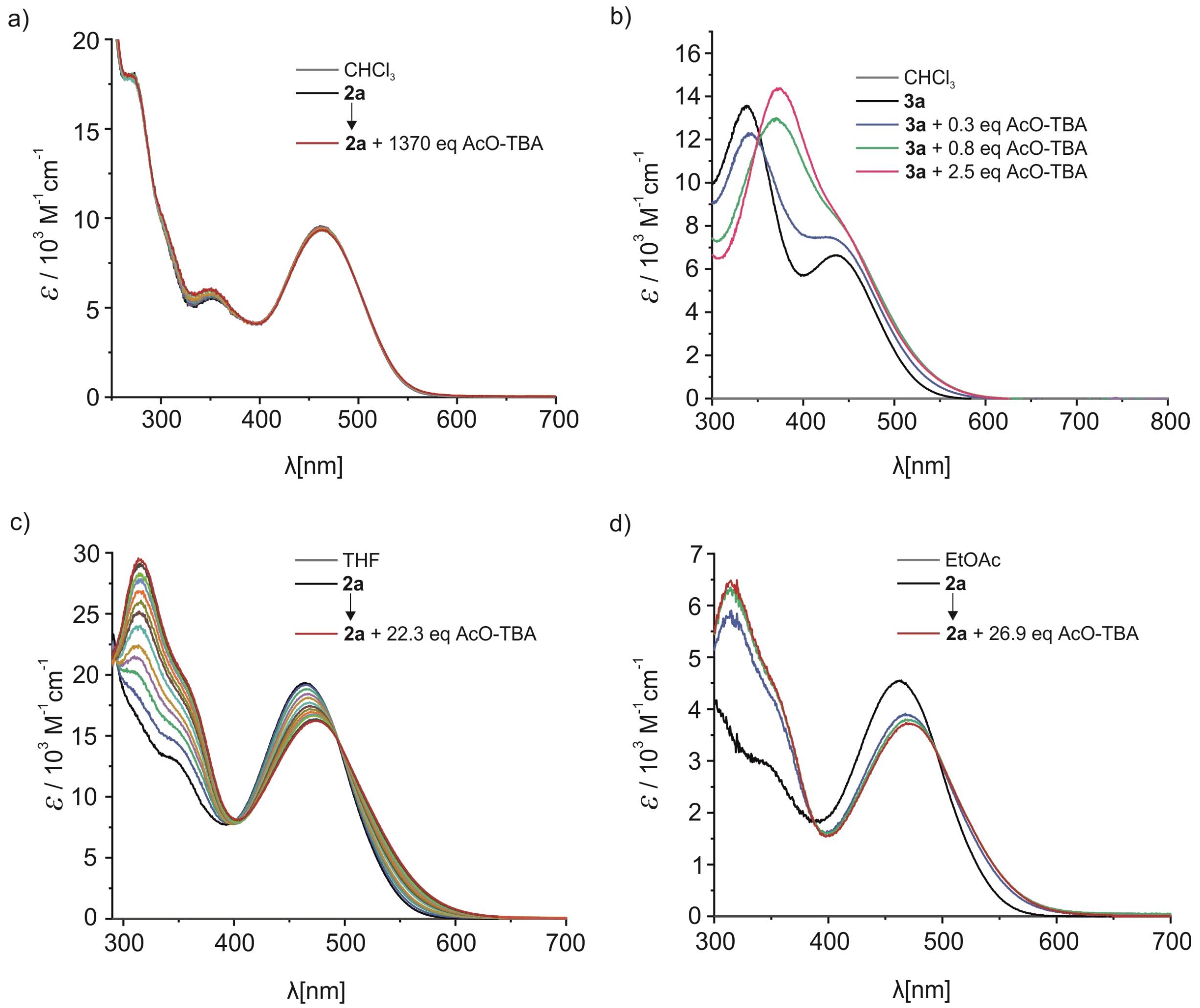

Figure S10. a) UV/Vis absorption spectra of a) **2a** ([**2a**] = 1.2 × 10^{-5} M) and b) **3a** ([**3a**] = 2.5 × 10^{-5} M) in $CHCl_3$, c) **2a** ([**2a**] = 1.2 × 10^{-5} M) in THF and d) **2a** ([**2a**] = 1.1 × 10^{-5} M) in EtOAc upon addition of tetrabutylamonium acetate (AcO-TBA).

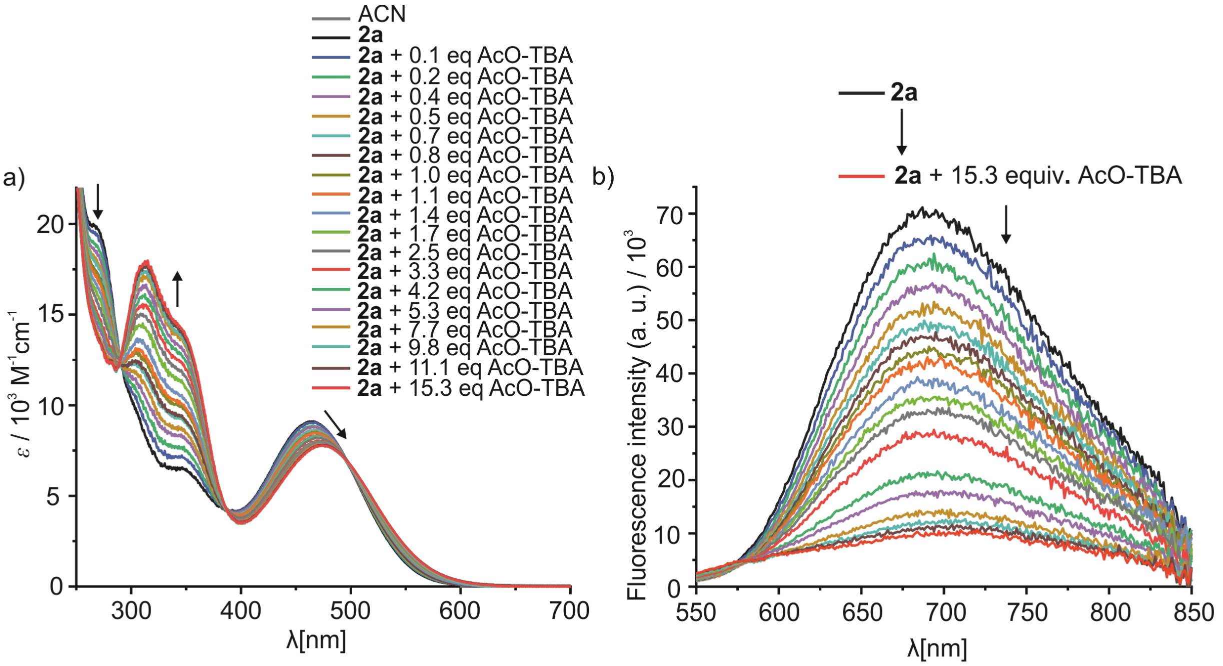

Figure S11. a) UV/Vis absorption and b) emission (λ_{ex} = 499 nm) spectra of **2a** ([**2a**] = 1.1 x 10^{-5} M) in ACN upon addition of tetrabutylamonium acetate (AcO-TBA). Virtually identical to Figure 3 and only reproduced here for better comparison with Figures S12–S14.

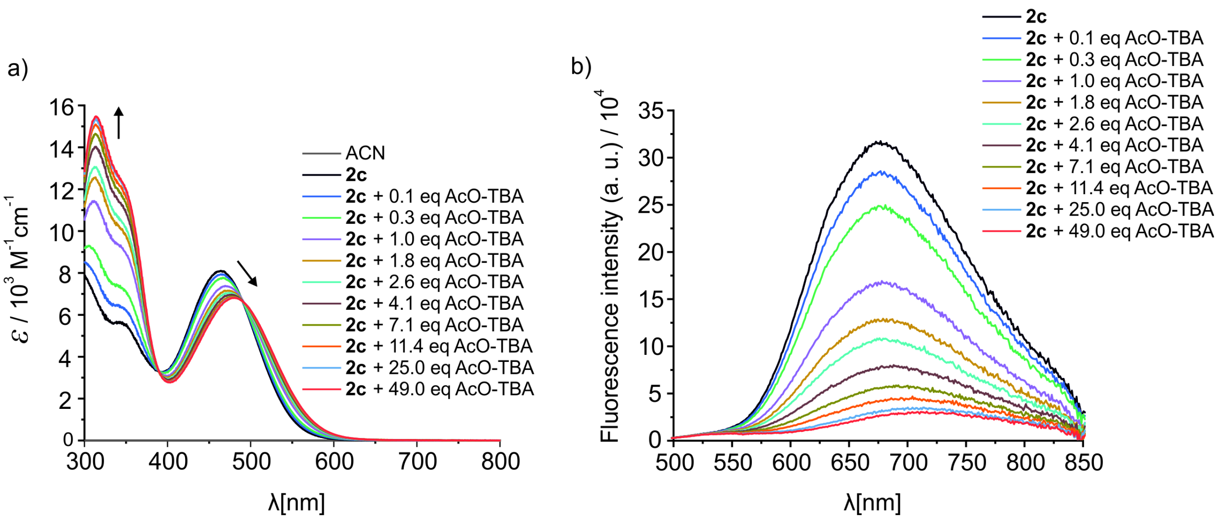

Figure S12. a) Absorption, b) emission (λ_{ex} = 488 nm) spectra of **2c** ([**2c**] = 1.0 x 10^{-5} M) in ACN upon addition of tetrabutylamonium acetate (AcO-TBA).

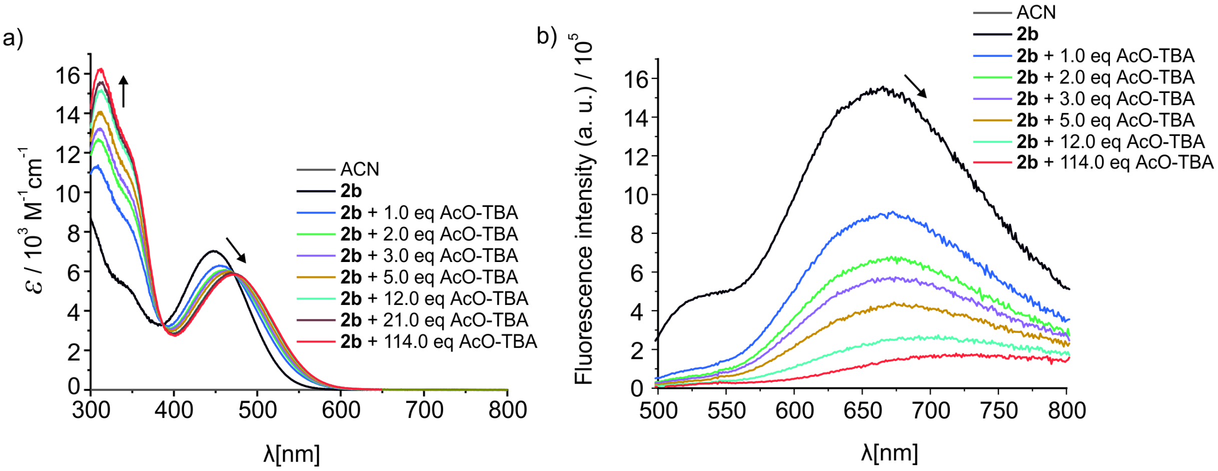

Figure S13. a) Absorption and b) emission (λ_{ex} = 470 nm) spectra of **2b** ([**2b**] = 1.4 x 10^{-5} M) in ACN upon addition of tetrabutylamonium acetate (AcO-TBA).

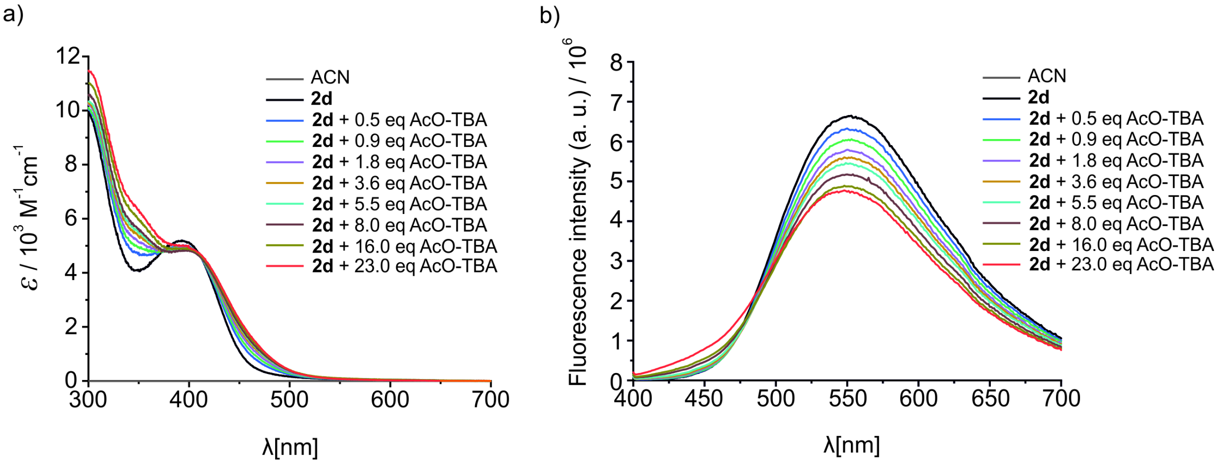

Figure S14. a) Absorption and b) emission (λ_{ex} = 390 nm) spectra of **2d** ([**2d**] = 2.4 x 10^{-5} M) in ACN upon addition of tetrabutylamonium acetate (AcO-TBA).

3. Molecularly imprinted polymer (MIP) synthesis

Preparation of CPDB-coated SiO_2 microparticles. Monodisperse, spherical SiO_2 particles with a diameter of 316 nm were prepared by the Stöber method,[5] adopted by us as reported previously.[6] Activation of the core particles with (3-aminopropyl)triethoxysilane (APTES) and RAFT (reversible addition–fragmentation chain transfer) agent 4-cyano-4-(phenylcarbonothioylthio)pentanoic acid (CPDB) functionalization for subsequent controlled RAFT polymerization was also carried out as described in reference.[6]

Template preparation. Enoxacin tetrabutylammonium (ENOX-TBA) was used as template for the synthesis of silica core-MIP shell particles. The template preparation was done by dissolving 160 mg TBA-OH x 30 H_2O in 8 ml ACN and adding an equimolar portion of this solution to the template. The solution was left in a thermomixer for 1 h (20°C, 900 rpm). Then, the template was concentrated under vacuum at 200 mbar for 15 min and at 0 mbar for 1 h and later overnight in a vacuum oven.

Preparation of fluorescent sensory non imprinted microparticles (NIPs). A mixture of **2c** with the co-monomer 2-hydroxyethyl methacrylate (HEMA) and the cross-linker ethylene glycol dimethacrylate (EGDMA) in a 60:40 ratio, respectively, was used for the synthesis of a non-imprinted polymer (NIP). Unfortunately, particles with a homogeneous polymer shell were not formed most probably due to the low solubility of the (non-complexed) fluorescent dye in ACN at mM concentrations.

4. Particle characterization: zeta potential and thermogravimetric analysis (TGA)

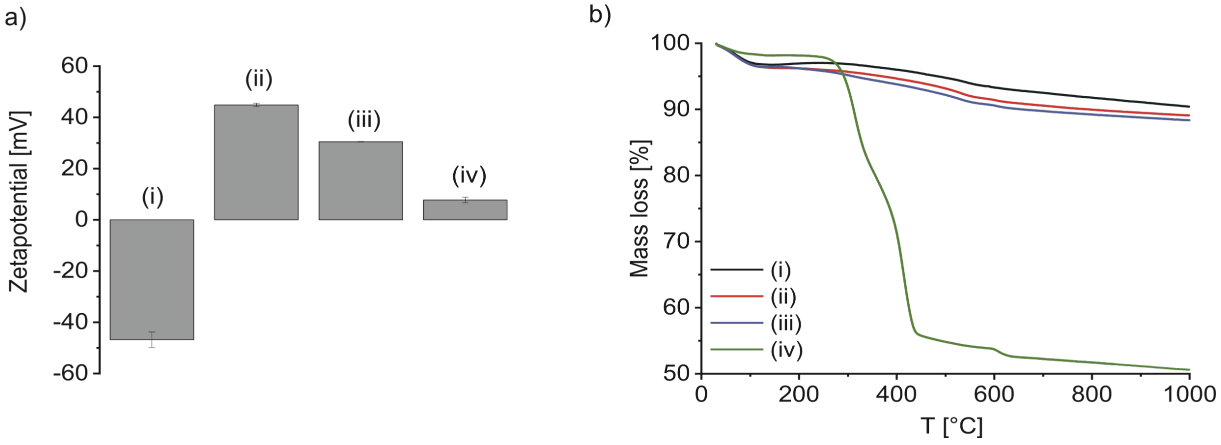

Figure S15 (a) Zeta potential measured at pH 6 and (b) TGA curves of (i) SiO_2 particles, (ii) APTES@SiO_2, (iii) RAFT@SiO_2 and (iv) MIPs.

5. Fluorescence measurements with MIP sensor particles in ACN

The binding properties of the MIPs were investigated by fluorescence measurements. 0.42 mg core-shell particles were suspended in 2 mL ACN in a 10 mm quartz cell. The suspension was titrated with 1 mM ENOX-TBA, AMPI-TBA or AMOX-TBA. After each step of addition, the suspension was equilibrated for 2 min before measuring the fluorescence. Plotting the reduced fluorescence intensity changes $\Delta F/F_0$ (F_0 = fluorescence intensity in absence of analyte; ΔF = fluorescence intensity change for each titration step) of the MIP particles against the concentration of the analyte allows assessment of the sensing performance. This normalization was important to avoid experimental influences related to a slight polydispersity of the microparticles.

6. TEM images

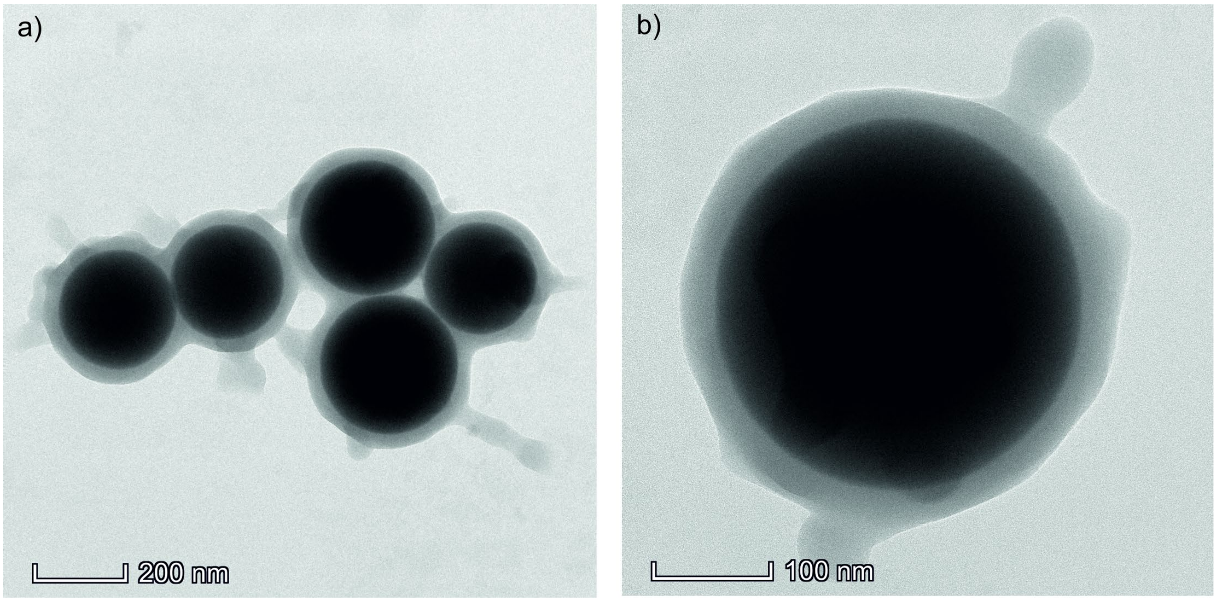

Figure S16 TEM images of ENOX-TBA imprinted core shell silica nanoparticles. Scale bar = 200 nm (a) and 100 nm (b).

7. Absorption spectra of 2c and 2c-containing ENOX-TBA-imprinted core-shell silica particles in ACN.

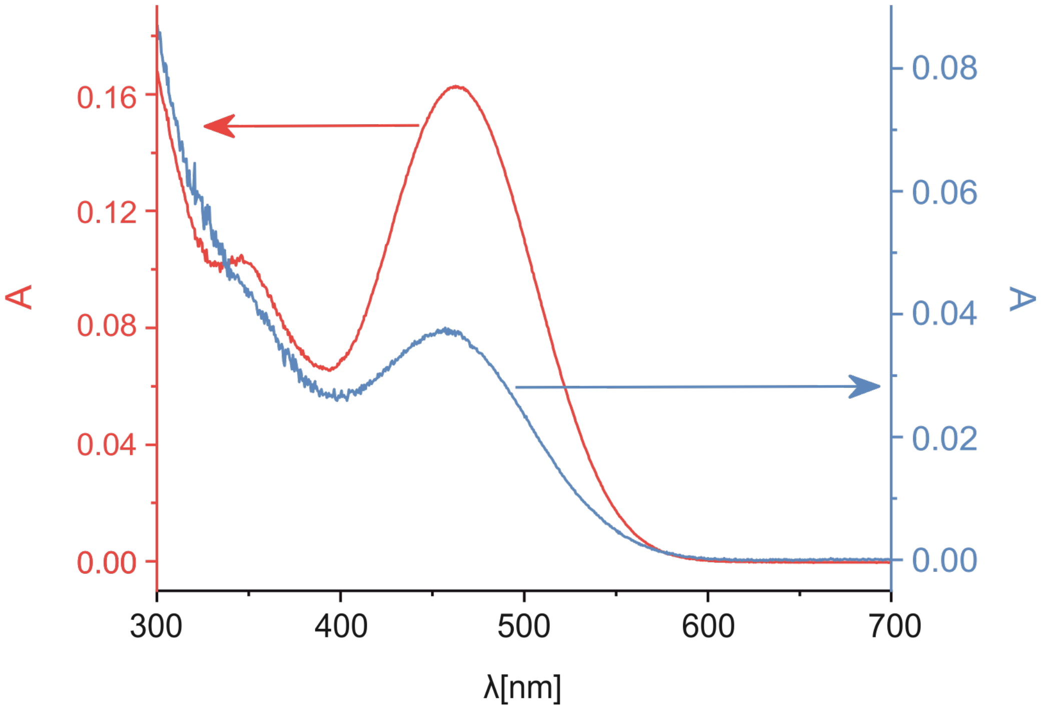

Figure S17 Absorption spectra of **2c** (red) and **2c**-containing ENOX-TBA-imprinted core-shell silica particles (blue) in ACN showing that **2c** remains stable under polymerization conditions.

8. Fluorescence spectra of MIP particles with AMOX-TBA and AMPI-TBA

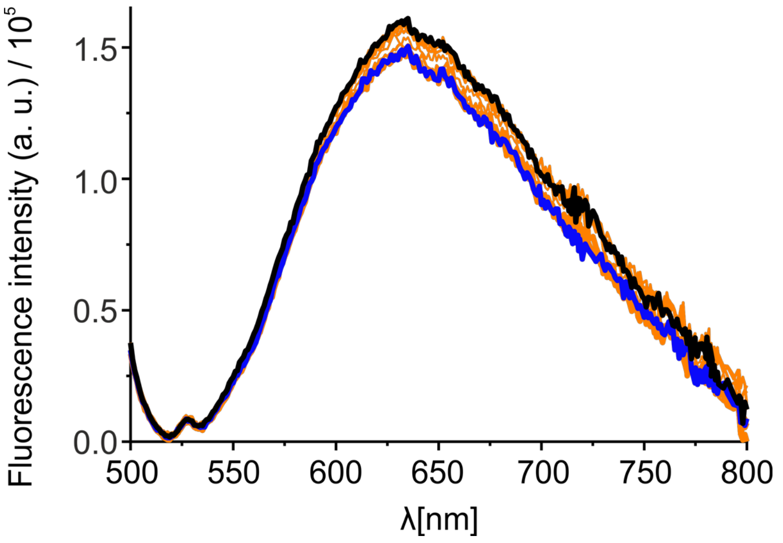

Figure S18. Fluorescence emission (λex = 480 nm) spectra of the sensory MIP developed for sensing ENOX-TBA in the absence (black line) and in the presence of AMOX-TBA up to 111 µM (blue line).

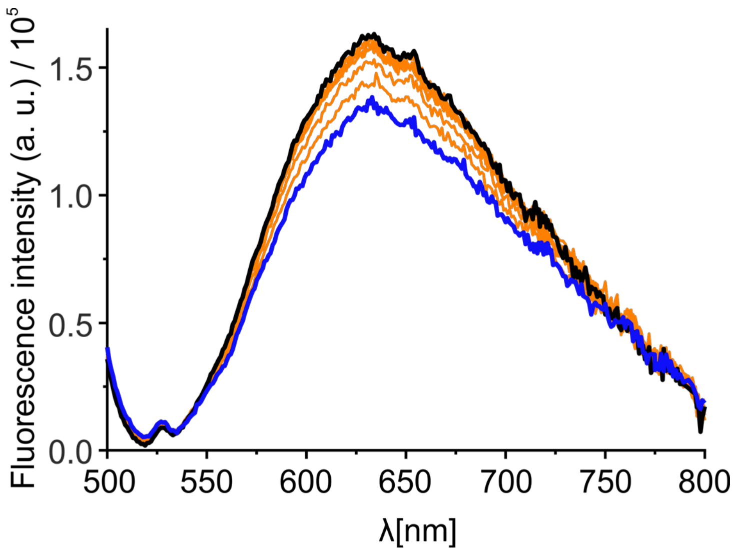

Figure S19. Fluorescence emission (λex = 480 nm) spectra of the sensory MIP developed for sensing ENOX-TBA in the absence (black line) and in the presence of AMPI-TBA up to 111 µM (blue line).

9. Uncertainty budget

$$u_{rel}^{\ 2} = n \cdot u_{rel}^{w\ 2} + p \cdot u_{rel}^{s\ 2} + q \cdot u_{rel}^{d\ 2} + u_{rel}^{a\ 2} + u_{rel}^{r\ 2}$$

Eq. S1 Total relative errors for each sample taking in account the errors coming from the different steps (see ref.[7] and [8] for details)

with
- n × weighing of the products: $u_{rel}^{w} = 0.02\%$
- p × dissolving the products: $u_{rel}^{s} = 1\%$
- q × dilution of stock solution: $u_{rel}^{d} = 1\%$
- Relative uncertainty of emission: $u_{rel}^{a} = 0.6\%$
- Repetition of the experiment (N = 3): $u_{rel}^{r} = 2.4\%$

10. References

[1] D. Brynn Hibbert and P. Thordarson, *Chem. Commun.* **2016**, *52*, 12792-12805.
[2] C. A. Schneider, W. S. Rasband and K. W. Eliceiri, *Nat. Methods* **2012**, *9*, 671-675.
[3] H. Matsunaga, T. Santa, T. Iida, T. Fukushima, H. Homma and K. Imai, *Analyst* **1997**, *122*, 931-936.
[4] I. Cummins, D. J. Wortley, F. Sabbadin, Z. S. He, C. R. Coxon, H. E. Straker, J. D. Sellars, K. Knight, L. Edwards, D. Hughes, S. S. Kaundun, S. J. Hutchings, P. G. Steel and R. Edwards, *Proc. Natl. Acad. Sci. USA* **2013**, *110*, 5812-5817.
[5] W. Stober, A. Fink and E. Bohn, *J. Colloid Interface Sci.* **1968**, *26*, 62-69.
[6] W. Wan, M. Biyikal, R. Wagner, B. Sellergren and K. Rurack, *Angew. Chem. Int. Ed.* **2013**, *52*, 7023-7027.
[7] K. Rurack and M. Spieles, *Anal. Chem.* **2011**, *83*, 1232-1242.
[8] S. Wagner, J. Bell, M. Biyikal, K. Gawlitza and K. Rurack, *Biosens. Bioelectron.* **2018**, *99*, 244-250.
